# Large-Scale Phenomics Identifies Primary and Fine-Tuning Roles for CRKs in Responses Related to Oxidative Stress

**DOI:** 10.1371/journal.pgen.1005373

**Published:** 2015-07-21

**Authors:** Gildas Bourdais, Paweł Burdiak, Adrien Gauthier, Lisette Nitsch, Jarkko Salojärvi, Channabasavangowda Rayapuram, Niina Idänheimo, Kerri Hunter, Sachie Kimura, Ebe Merilo, Aleksia Vaattovaara, Krystyna Oracz, David Kaufholdt, Andres Pallon, Damar Tri Anggoro, Dawid Glów, Jennifer Lowe, Ji Zhou, Omid Mohammadi, Tuomas Puukko, Andreas Albert, Hans Lang, Dieter Ernst, Hannes Kollist, Mikael Brosché, Jörg Durner, Jan Willem Borst, David B. Collinge, Stanisław Karpiński, Michael F. Lyngkjær, Silke Robatzek, Michael Wrzaczek, Jaakko Kangasjärvi, on behalf of the CRK Consortium

**Affiliations:** 1 The Sainsbury Laboratory, Norwich Research Park, Norwich, United Kingdom; 2 Department of Plant Genetics, Breeding and Plant Biotechnology, Warsaw University of Life Sciences-SGGW, Warsaw, Poland; 3 Department of Biosciences, Plant Biology, University of Helsinki, Helsinki, Finland; 4 Laboratory of Biochemistry and Microspectroscopy Center, Wageningen University, Wageningen, The Netherlands; 5 Department of Plant and Environmental Sciences and Copenhagen Plant Science Center, University of Copenhagen, Frederiksberg, Denmark; 6 Institute of Technology, University of Tartu, Tartu, Estonia; 7 Department of Plant Physiology, Warsaw University of Life Sciences-SGGW, Warsaw, Poland; 8 Research Unit Environmental Simulation, Helmholtz Zentrum München, German Research Center for Environmental Health, Neuherberg, Germany; 9 Institute of Biochemical Plant Pathology, Helmholtz Zentrum München, German Research Center for Environmental Health, Neuherberg, Germany; 10 Distinguished Scientist Fellowship Program, College of Science, King Saud University, Riyadh, Saudi Arabia; University of Minnesota, United States of America

## Abstract

Cysteine-rich receptor-like kinases (CRKs) are transmembrane proteins characterized by the presence of two domains of unknown function 26 (DUF26) in their ectodomain. The CRKs form one of the largest groups of receptor-like protein kinases in plants, but their biological functions have so far remained largely uncharacterized. We conducted a large-scale phenotyping approach of a nearly complete *crk* T-DNA insertion line collection showing that CRKs control important aspects of plant development and stress adaptation in response to biotic and abiotic stimuli in a non-redundant fashion. In particular, the analysis of reactive oxygen species (ROS)-related stress responses, such as regulation of the stomatal aperture, suggests that CRKs participate in ROS/redox signalling and sensing. CRKs play general and fine-tuning roles in the regulation of stomatal closure induced by microbial and abiotic cues. Despite their great number and high similarity, large-scale phenotyping identified specific functions in diverse processes for many CRKs and indicated that CRK2 and CRK5 play predominant roles in growth regulation and stress adaptation, respectively. As a whole, the CRKs contribute to specificity in ROS signalling. Individual CRKs control distinct responses in an antagonistic fashion suggesting future potential for using CRKs in genetic approaches to improve plant performance and stress tolerance.

## Author Summary

Receptor-like kinases (RLKs) are important regulators in signal transduction in plants. However, the large number of RLKs and their high sequence similarity has hampered the analysis of RLKs. One of the largest subgroups of RLKs, the cysteine-rich receptor-like kinases (CRKs), has been suggested to be involved in mediating the effects of reactive oxygen species (ROS). While ROS are recognized as important signalling elements with a large variety of roles in plants, their ligands and achievement of signalling specificity remain unknown. Using insertion mutants we analysed the roles of CRKs in plant development and stress responses and show that CRKs have important roles as mediators of signalling specificity during regulation of stomatal aperture. Our study shows that, despite their large number and high sequence conservation, individual CRKs have intriguingly distinct functions in different aspects of plant life. This makes the CRKs promising candidates for future studies of their biochemical function.

## Introduction

Receptor protein kinases play key roles in mediating perception of extracellular signals. These signals trigger intracellular signalling cascades allowing cells to respond and adapt to internal and external stimuli. Receptor kinases contain an extracellular signal-sensing domain connected by a single transmembrane domain to an intracellular protein kinase domain [1]. During evolution, different systems for the same function have emerged in animals and plants: animals deploy receptor-tyrosine kinases whereas plants utilize receptor-like kinases (RLKs), which are dual specificity serine/threonine and tyrosine kinases [2–4]. In contrast to mammals, all sequenced plant genomes contain a large number of RLKs, as illustrated by *Arabidopsis* and rice which encode more than 600 and 1100 RLKs in their genomes [1], respectively. The highly diverse extracellular regions of RLKs typically contain one or more protein domains or combinations of different domains. These domains have been used to divide RLKs into different sub-groups [1]. To date only a few RLKs have been functionally characterised but expression analyses have linked a large number of RLKs to many different physiological processes and signalling networks in plant development, pathogen defence, and abiotic stress response [5–8].

The cysteine-rich receptor-like kinases (CRKs, originally referred to as domain of unknown function 26 [DUF26] RLKs [9]) represent one of the largest groups of RLKs with 44 members in *Arabidopsis thaliana* [7]. Most CRKs have a typical RLK domain architecture, but three CRKs (CRK43, CRK44 and CRK45) consist only of the cytoplasmic domain reminiscent of receptor-like cytoplasmic kinases (RLCKs) [1]. The extracellular domain of CRKs encompasses two copies of the DUF26 domain (PF01657; http://pfam.sanger.ac.uk/family/PF01657; stress-antifung domain), which contains three cysteine residues in a conserved configuration (C-X_8_-C-X_2_-C) and is a predicted target for redox modifications. The DUF26 domain is also present in eight *Arabidopsis* PLASMODESMATA-LOCATED PROTEINs (PDLPs) [10]. The domain structure of these PDLPs is similar to CRKs but lacks the intracellular protein kinase, analogous to the leucine-rich repeat (LRR) receptor-like proteins (RLPs). Experimental evidence suggests that PDLPs are involved in the regulation of cell-to-cell communication and are important for pathogen defence [11,12]. Furthermore, more than 50 secreted proteins in *Arabidopsis* contain DUF26 domains but their roles have so far not been elucidated.

Several *CRK*s show elevated transcript levels in response to salicylic acid (SA) and pathogens [13–17] as well as ozone (O_3_) and drought [7,18]. Altered transcript abundance due to conditions affecting cellular redox and reactive oxygen species (ROS) balance [7,19], and the presence and spacing of the conserved cysteines in the DUF26 domain suggest that CRKs might be connected to ROS and redox signalling [7,14,20]. However, the functional role of the DUF26 domain is still unclear.

Previous studies have suggested the involvement of some CRK family members in pathogen defence and osmotic stress. Overexpression of *CRK4*, *CRK5*, *CRK19*, and *CRK20* induced hypersensitive response-like (HR-like) cell death [13,14] and overexpression of *CRK4*, *CRK6*, *CRK13*, and CRK36 resulted in enhanced tolerance to the bacterial pathogen *Pseudomonas syringae* pv. *tomato* DC3000 (*Pto* DC3000) [21,22]. Also a loss-of-function mutant *crk20* showed a slight reduction in *Pto* DC3000 growth [23]. A *Medicago truncatula* CRK, SymCRK, was found to be involved in preventing early senescence and defence responses during symbiotic interactions [24]. Knock-down of *CRK36* resulted in increased sensitivity to abscisic acid (ABA) and osmotic stress [25], and *altered seed germination 6* (*asg6; crk2*), a mutant deficient in CRK2 function, has been associated with changes in seed germination in response to ABA [26]. A mutation in *crk7* led to slightly increased sensitivity to extracellular ROS [27], and a mutation in *crk5* resulted in impaired stomatal conductance, accelerated senescence as well as enhanced cell death in response to ultraviolet radiation [28]. Given the large number of CRKs and several transcript profiling experiments which suggest that CRKs are involved in a variety of environmental responses [6–8,18,19], it is surprising how little is known about the physiological and biochemical functions of this RLK family. However, based on transcriptional analysis CRKs might have far more complex functions, for example also in signalling in response to *N-*acetylglucosamine (GlcNAc) oligomers in the plant cell wall [29].

In this study we describe a comprehensive phenotypic analysis of a T-DNA insertion collection for the entire *CRK* gene family. This phenomics approach revealed novel roles for CRKs in control of plant development and biotic and abiotic stress adaptation. In spite of high amino acid sequence similarity, we observed that many CRKs mediate specific functions, with CRK2 and CRK5 playing predominant roles in growth regulation and stress adaptation, respectively. Our results imply a model for CRK function, placing CRKs as putative elements between ROS production and downstream signalling leading to pathogen- and abiotic stress-induced stomatal closure. This provides a framework for future detailed analysis of the molecular mechanisms underlying CRK signalling.

## Results

### Compilation and curation of a T-DNA insertion collection for the CRK group of RLKs

**Fig 1:**
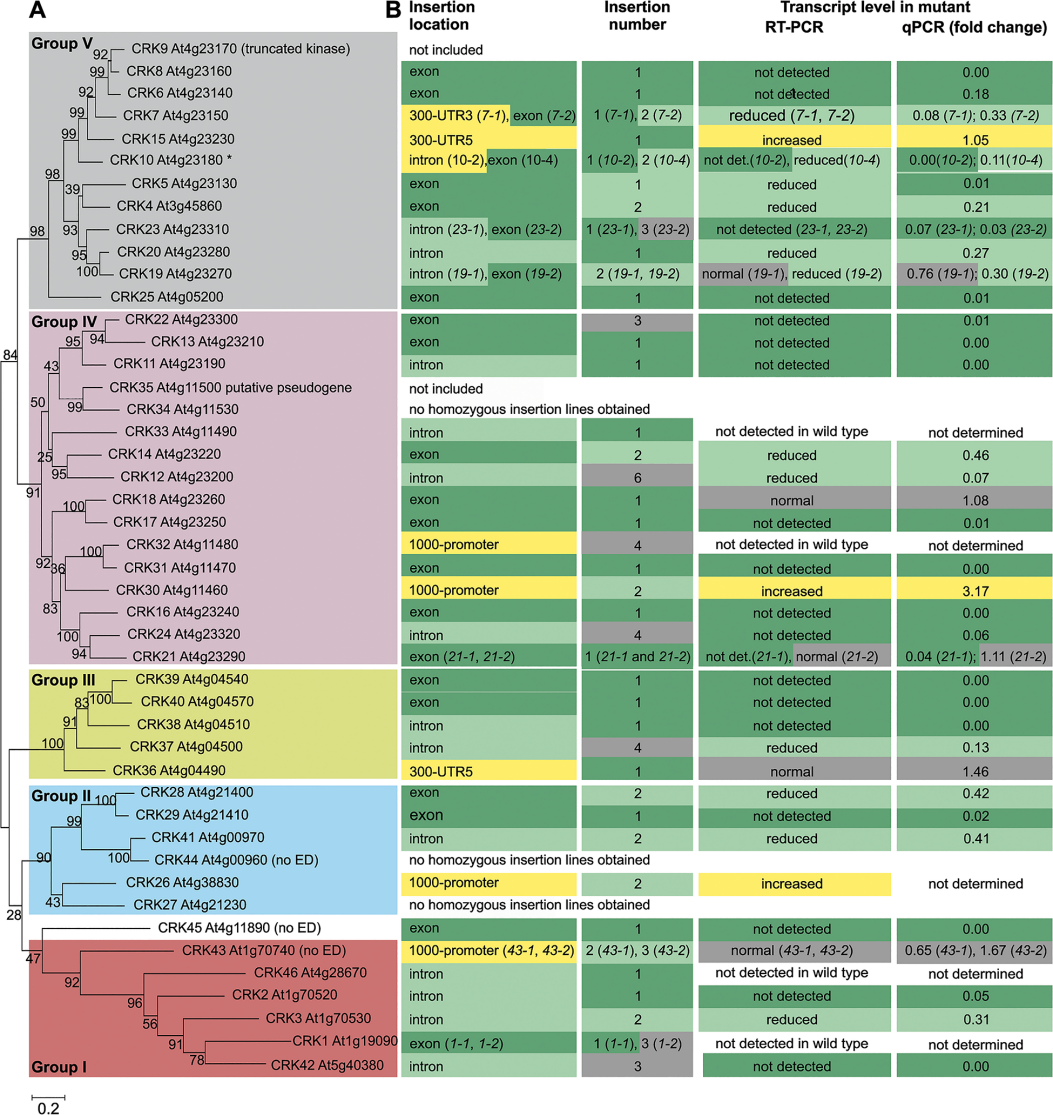
Phylogenetic clustering of the *Arabidopsis thaliana* CRK group of RLKs and summary of the *crk* T-DNA insertion collection. **(A)** The coding region of the CRKs of *Arabidopsis thaliana* (including the truncated *CRK9 At4g23170* and the putative pseudogene *CRK35 At4g11500*) was aligned using Muscle. The maximum-likelihood phylogenetic tree was estimated in MEGA6 using all sites (no gap penalty). The initial guide tree was constructed using maximum parsimony. Values at branch nodes represent bootstrap values (1000 replicates). CRK43 (At1g70740), CRK44 (At4g00960) and CRK45 (At4g11890) lack signal peptide, CRK ectodomain (ED) and transmembrane domain. **(B)** Information on T-DNA insertion lines for corresponding *crk* mutants is summarized: location of the T-DNA insertion in the gene (detailed information in S3 Fig), number of T-DNA insertions per line (determined by quantitative PCR; S1 Table) and transcript level of the corresponding *crk* mutant (according to semi-quantitative RT-PCR and qPCR; detailed information in S1 Table). For two additional *crk10* alleles (*crk10-1* and *crk10-3*) information can be found in S1 Table.

Transcriptional analyses have shown that *CRK* genes are responsive to several external stimuli. However, only limited information is available on their physiological roles. Based on the amino acid sequences of the coding region, the 44 *Arabidopsis thaliana* CRKs (plus the putative pseudogene *CRK35 At4g11500* and the truncated *CRK9 At4g23170*) form five distinct groups (Fig 1A). Similar groups can be identified in phylogenetic trees based on the intracellular kinase domain (S1A Fig) as well as on the extracellular region (S1B Fig). This suggests co-evolution of the extra- and intracellular domains of *Arabidopsis* CRKs. A group of six CRKs, group I, constitutes a basal clade which forms a sister group distinct from groups II-V. The position of CRK45, At4g11890, which lacks the extracellular and transmembrane region, is ambiguous as it clusters with low bootstrap support with the basal group in the phylogenetic tree based on the entire coding region (Fig 1A) but as a sister to groups II and III in the tree based on the kinase domain (S1A Fig). Thus, CRK45 is not assigned to any CRK subgroup. Group I *CRKs* are distributed across chromosomes 1, 4 and 5 and only *CRK2* and *CRK3* are located next to each other, whereas genes encoding *CRKs* in groups II-V are, with the exception of *CRK4* on chromosome 3, located on chromosome 4 and organized in repeats forming clusters of CRK genes (S2 Fig).

**Fig 2:**
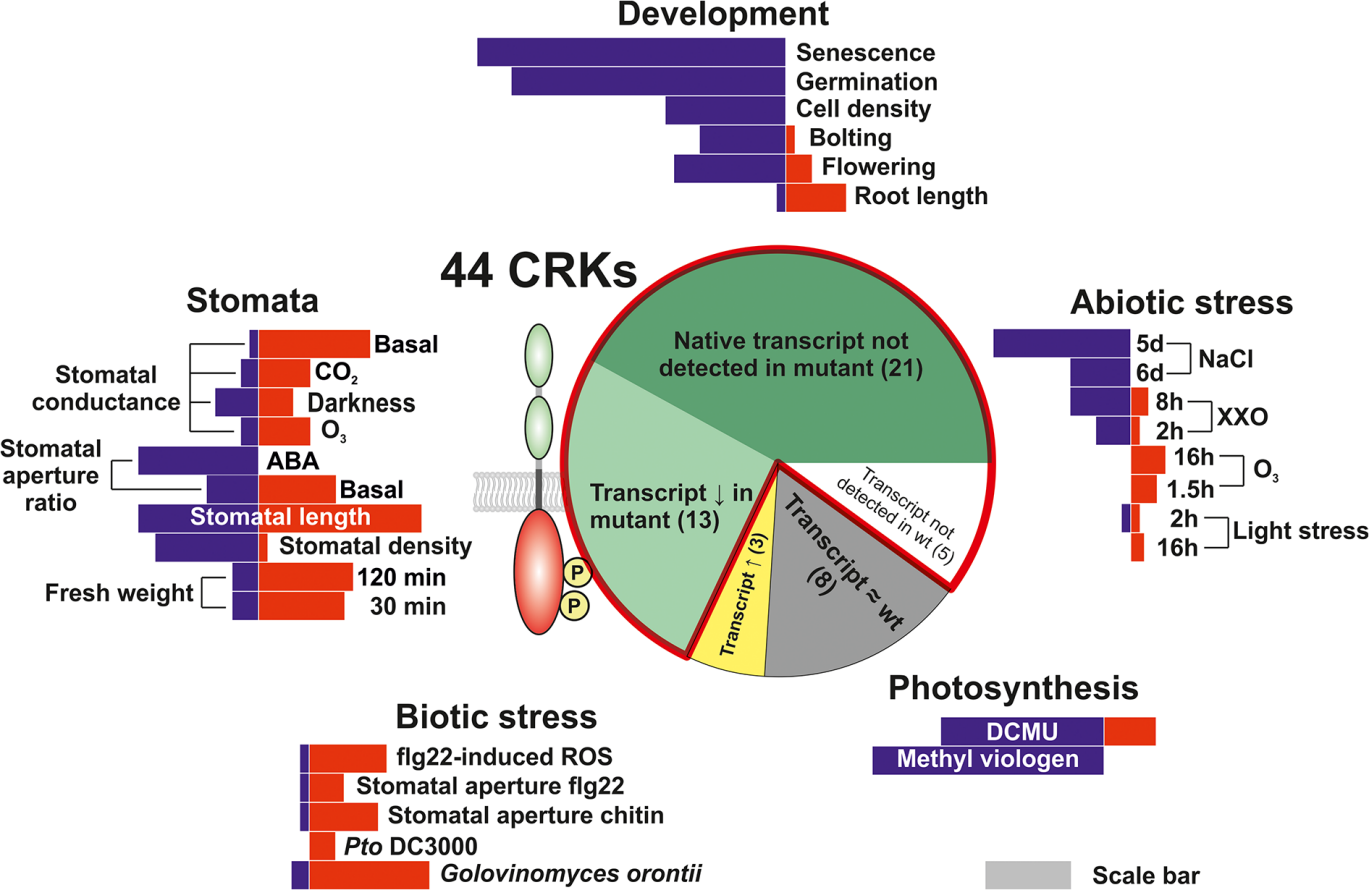
Phenotypic analysis of the *Arabidopsis thaliana* CRK protein family. A T-DNA insertion collection for the CRK family was compiled and subjected to phenotyping addressing aspects of plant development, biotic and abiotic stress responses, photosynthesis as well as stomatal regulation. Length of red and blue bars in the five phenotyping sections is representative of the number of *crk* lines found to have phenotypes in the thematic area. Information about the sections in the pie chart is displayed in Figs 1 and S3 and S4 and S1 Table. The red outline in the pie chart highlights the lines included in the analyses and figures throughout the manuscript. The gray scale bar serves as a reference for comparison. The length of the scale bar corresponds to ten lines.

In order to investigate the function of individual CRK family members, a collection of 82 T-DNA insertion mutants was compiled from the Nottingham Arabidopsis Stock Centre (NASC; Figs 1B and S3 and S4 and S1 Table). *CRK35* (putative pseudogene) and *CRK9* (truncated) were excluded and, under our conditions, no homozygous T-DNA insertion lines could be obtained for *crk27*, *crk34*, and *crk44* in the Col-0 background (Figs 1B and S3 and S1 Table). A total of 50 homozygous T-DNA insertion lines representing 41 *crk* mutants were isolated. In 21 *crk* lines the corresponding wild-type *CRK* transcript was not detected while levels were reduced in thirteen lines (Figs 1B and S4 and S1 Table). Of those thirteen lines seven have an insertion in an exon suggesting that they would produce a truncated protein while five lines carried insertions in introns. Only one line, *crk7-1*, with reduced transcript level of a *CRK* carried an insertion in the 5’ untranslated region (UTR). The status of the *crk1-1*, *crk1-2*, *crk32*, *crk33*, and *crk46* mutants is unclear (Figs 1B and S3 and S1 Table) since transcripts for those four *CRKs* (*CRK1*, *CRK32*, *CRK33*, *CRK46*) were not detectable in the Col-0 wild type. In three *crk* lines where insertions were located in the 5’ UTR or the upstream promoter region, the corresponding *CRK* transcript abundance was increased compared to wild type. In eight lines no differences in corresponding *CRK* transcript levels were detected between the T-DNA insertion line and wild type (Figs 1B and S4 and S1 Table). Of those lines, two carried the insertions in the exonic region, thus possibly resulting in a truncated protein; one line carried the insertion in an intron, while the remaining five lines carried insertions in the regions upstream of the start codon (S3 Fig). The eleven lines in which corresponding *CRK* transcript levels were increased or not altered compared to Col-0 wild type were excluded from subsequent analyses (Fig 2). Thus a total of 39 lines were used (Fig 2). Based on quantitative PCR (qPCR) analysis 27 of the T-DNA insertion lines contained a single insertion, twelve contained two insertions while the rest contained more than two insertions in their genomes (Fig 1B and S1 Table). An age-matched seed collection was generated and used for all subsequent phenotypic analyses where we investigated the role of CRKs in aspects of growth/development, abiotic stress responses, biotic stress responses, photosynthesis and stomatal regulation (Fig 2 and S2 Table).

### Development is altered in *crk* mutants

**Fig 3:**
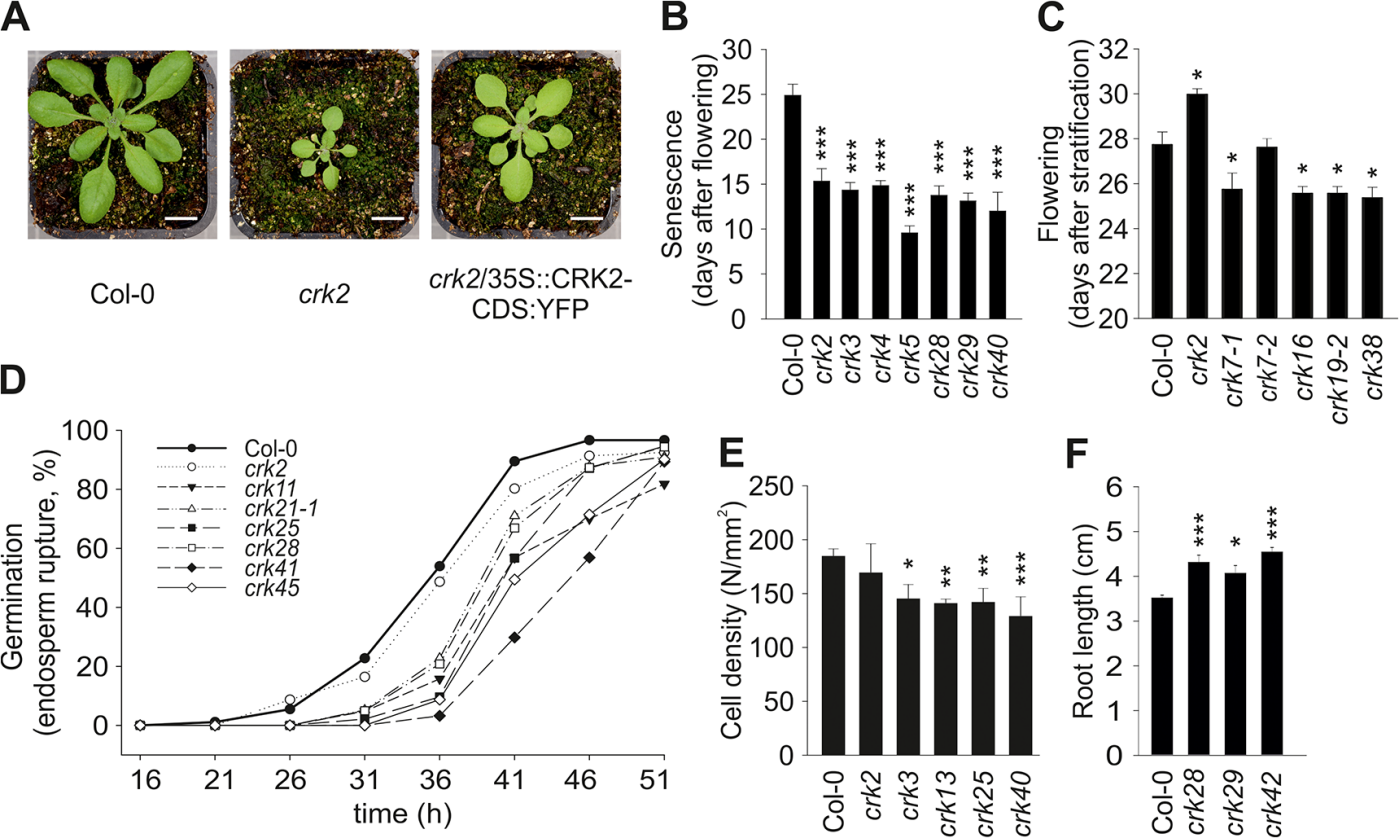
Plant development is affected in several *crk* mutants. **(A)** Representative pictures of 17-day old seedlings of Col-0 wild type and *crk2*. Complementation of *crk2* with 35S::*CRK2-CDS*:YFP rescued the growth defect of the mutant. Plants were grown under the following conditions: 250 μmol m^-2^ s^-1^ light intensity under 12 h-day length (day: 23°C, 70% relative humidity; night: 18°C, 90% relative humidity). Bar = 1 cm. Pictures are representative of three independent experiments. **(B)** A selection of *crk* mutant lines showing earlier senescence compared to Col-0 wild type. Results are means ± SE (*n* = 8). **(C)** Several *crk* mutants flowered earlier compared to wild type while *crk2* flowered later. Results are means ± SE (*n* = 8). **(D)** Time course analysis of endosperm rupture showed delayed germination in several *crk* mutants compared to wild type. Results represent means from three independent biological experiments (*n* = 30). Testa and endosperm rupture were assessed every 5 hours up to 51 hours of imbibition. A seed was considered as germinated when the radicle protruded through both envelopes. **(E)** Several *crk* mutants exhibit a lower pavement cell density (number of pavement cells / mm^2^) in cotyledons. Results are means ± SE (*n* = 15). **(F)** Three *crks* showed slightly longer roots compared to wild type (measured eight days after stratification). Results are means ± SE (*n* = 16). (**B-F)** Differences between mutants and Col-0 wild type were compared and analysed using one-way-ANOVA (*post hoc* Dunnett, asterisks indicate statistical significance at *P<0.05, **P<0.01 and ***P<0.001) for **(B, C, E**) and linear model with single step p-value adjustment (**F**). All experiments were repeated three times with similar results.

Most *crk* mutants displayed normal morphology similar to the Col-0 wild type (S5 Fig). By contrast, *crk2* displayed a clear dwarf phenotype (Fig 3A). Complementation of the *crk2* mutation restored morphology similar to the Col-0 wild type (Fig 3A). The *crk5* mutant was slightly smaller compared to Col-0 wild type, in particular after five weeks of growth [28], and overexpression of CRK5 in the mutant background led to slightly larger rosettes. Under long day conditions (16h light/8h darkness) 34 *crk* mutants displayed early senescence (Figs 3B and S6A). Under the same conditions, four *crk* mutants flowered earlier than Col-0 wild type (Figs 3C and S6B). The only mutant that flowered later compared to Col-0 wild type was the dwarf *crk2* (Fig 3C). Germination was delayed in 32 *crk* mutants as shown by analysis of endosperm rupture (Fig 3D and S3 Table). Pavement cell density was reduced in eleven *crks* compared to Col-0 wild type (Figs 3E and S6C). Germination and pavement cell density were not altered in the dwarf *crk2* compared to Col-0 wild type (Fig 3D and 3E). In addition, *crk28*, *crk29*, and *crk42* showed slightly longer roots compared to Col-0 wild type seedlings (Fig 3F).

In summary, our results suggest that the CRKs are not exclusively involved in stress and pathogen responses, as previously suggested, but in addition contribute to the regulation of specific developmental processes. The only mutant displaying a clear dwarf phenotype was *crk2* which supports the earlier predictions that CRK2 might be involved in growth regulation in response to ABA [26].

### Responses of *crk* mutants to abiotic stresses

Elevated production of ROS is part of the response to many abiotic stresses [20]. *CRK* transcript levels were strongly regulated in response to abiotic stresses (S7 Fig) [7,19] including ozone (O_3_) and ultraviolet radiation (UV). O_3_-induced ROS formation in the extracellular space rapidly induced transcript accumulation for several *CRKs*, while high light-induced ROS formation in the chloroplasts showed no effect [7]. In line with this, high light stress did not induce extensive damage in the *crk* mutants (S8 Fig). Only *crk2* and *crk45* showed significant light stress-induced electrolyte leakage that differed from the response in wild type plants after nine or 24 hours, respectively.

**Fig 4:**
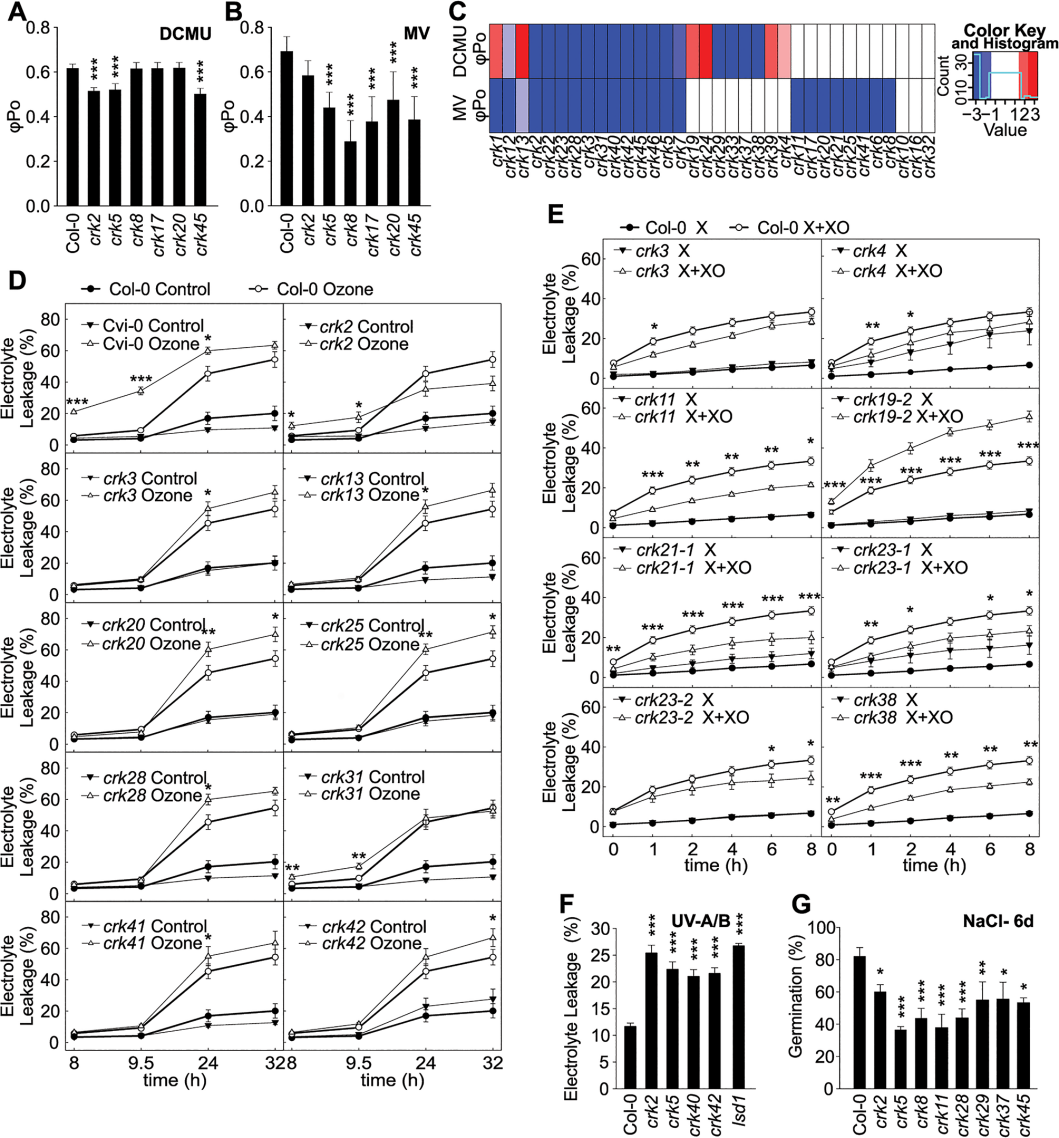
Abiotic stress responses are affected in *crk* mutants. **(A and B)** Treatment with 3-(3,4-dichlorophenyl)-1,1-dimethylurea (DCMU) for two hours **(A)** or methyl viologen (MV) for 48 hours **(B**) resulted in stronger impairment of photosynthesis in a subset of *crk* mutants compared to Col-0 wild type as shown by changes in the maximum quantum yield of primary photochemistry (*φ*_Po_). Significant differences in relation to wild type are indicated (*n* = 12, bars represent SD; one-way ANOVA with *post hoc* Tukey HSD) *** *P*<0.001. The experiment was repeated three times with similar results. **(C)** Clustering of DCMU and MV experiments. Red and blue indicate increased or decreased response with respect to Col-0, respectively (average of alleles). Color intensity is proportional to a Benjamini-Hochberg false discovery rate (FDR) adjusted Z statistic, which takes the estimated means and their variation into account. Roughly, |Z|>2 corresponds to an FDR<5%, and |Z|<2.6 to an FDR<1%. Results were clustered using a complete linkage algorithm with 1-Pearson correlation as distance. **(D)** In several *crk* mutant lines exposure to O_3_ resulted in elevated electrolyte leakage. Electrolyte leakage was measured at indicated time points after start of the O_3_ exposure that lasted 6 h (350 ppb). Mean values ± SD from two independent experiments are presented (*n* = 12, one-way ANOVA with *post hoc* Tukey HSD). **(E)** Xanthine-Xanthine Oxidase (X+XO) infiltration induced different levels of electrolyte leakage in *crk* mutants compared to wild type (0.1 U ml^-1^, 4 h). Mean values ± SD from three independent experiments are presented (*n* = 16, linear model with single-step p-value adjustment for multiple testing). (**F**) Treatment with ultraviolet-A (UV-A) and–B (UV-B) radiation led to increased electrolyte leakage in *crks* compared to wild type. Mean values ± SD from three independent experiments are presented (*n* = 12, one-way ANOVA with *post hoc* Tukey HSD). **(G)** Effect on NaCl on the germination *crk* seedlings at 6 days after stratification. Values represent mean of the ratio (germination percentage on 120 mM NaCl / percentage on control medium) for each line (*n* = 15, linear model, single-step p-value adjustment). Experiments were performed three times. Asterisks indicate statistically significant differences between *crks* and Col-0 (*P<0.05, **P<0.01 and ***P<0.001) (**D-G)**. Error bars indicate ± SE (**D, E** and **F)**. Relative electrolyte leakage was calculated as a ratio of the value measured at the indicated time and the total electrolyte leakage after freezing (**D and E**) or autoclaving the samples (**F**).

Elevated ROS production in chloroplasts can also be induced by methyl viologen (MV, also known as Paraquat), which leads to increased superoxide production in the reducing end of the PSII. Moreover, chloroplastic ROS formation can also be induced by 3-(3,4-dichlorophenyl)-1,1-dimethylurea (DCMU), which causes increased production of singlet oxygen by affecting the redox status of the plastoquinone pool. The use of MV or DCMU allowed the assessment of the *crk* responses to increased chloroplastic ROS production by measurement of photosynthetic energy transfer, which is a more sensitive measurement compared to electrolyte leakage. Treatment of plants with MV or DCMU resulted in stronger photoinhibition in several lines (*e*.*g*. *crk2*, *crk5*, *crk8*, *crk17*, *crk20*, *crk40*, *crk45*) compared to wild type, depending on the ROS inducer that was used (Figs 4A–4C and S9). Photosynthetic impairment of the *crk5* mutant was rescued by overexpression of CRK5 [28].

In summary, these results suggest that some CRKs are involved in sensing or adaptation to changes in ROS or redox balance in the chloroplast and could be involved in signal transduction processes that culminate in regulation of photosynthetic electron transport. Like most RLKs, CRKs are predicted to localize to the plasma membrane and no CRKs have been identified in studies exploring the chloroplast proteome [30,31]. Thus, at the moment it is not clear how the CRKs communicate with the chloroplast but they might participate in communication between apoplast and chloroplast similarly to what has been described during PAMP-triggered immunity [32].

Even though chloroplasts and peroxisomes are the main sources of intracellular ROS in plants, extracellular ROS production is also important in the response abiotic stimuli [20]. Extracellular ROS production can be specifically induced by exposure to the air pollutant O_3_ or infiltration with the enzymatic system Xanthine-Xanthine Oxidase (X+XO) [33]. Several *crk* mutants displayed differential responses to extracellular ROS in comparison to Col-0 wild type plants. Responses to O_3_ were generally subtle (Figs 4D and S10). At 9.5 hours after the onset of O_3_ treatment *crk2* and *crk31* showed increased electrolyte leakage while at later timepoints *crk3*, *crk13*, *crk20*, *crk25*, *crk28*, *crk41*, and *crk42*, showed elevated electrolyte leakage (Fig 4D). Enhanced O_3_-induced cell death, visible as lesion formation, was observed for *crk2*, *crk3*, *crk5*, *crk7-1*, *crk7-2*, *crk11*, *crk13*, *crk19-2*, *crk20*, *crk22*, *crk23-2*, *crk24*, *crk28*, *crk31*, *crk37*, *crk38*, and *crk46* (S11 Fig). In response to treatment with X+XO, *crk19-2* showed increased electrolyte leakage while *crk4*, *crk11*, *crk21-1*, *crk23-1*, *crk23-2*, *crk29*, *crk37*, *crk38* and *crk46* showed reduced electrolyte leakage compared to Col-0 wild type (Figs 4E and S12).

In response to UV-A and-B, *crk2*, *crk5*, *crk40*, and *crk42* displayed significantly elevated electrolyte leakage compared to Col-0 wild type indicating more damage similar to *lesion simulating disease 1* (*lsd1*), which was used as positive control (Figs 4F and S13A). Complementation of *crk5* rescued the hypersensitivity to UV-A and–B radiation [26]. Exposure to salt (NaCl) is another environmentally relevant abiotic stress. Overall, twenty one *crk* mutants, including *crk2*, *crk5*, *crk8*, *crk11*, *crk28 crk29 crk37*, and *crk45*, showed delayed germination compared to the Col-0 wild type on medium containing 120 mM NaCl (Figs 4G and S13B and S1C). After six days of growth on medium supplemented with 120 mM NaCl 13 *crk* mutants still maintained the delayed germination (S13C Fig).

While previous studies have emphasized the roles of CRKs in the response to pathogens and cell death regulation [13,14,21–23], our observations suggest that CRKs are also important regulators of the response to abiotic stresses, such as UV-A and–B, salt, and O_3_, possibly through extracellular ROS. In addition, our results suggest that the CRKs could be involved in controlling processes that indirectly regulate photosynthetic electron transport in the chloroplast.

### Stomatal development and regulation is altered in the *crk* mutants

**Fig 5:**
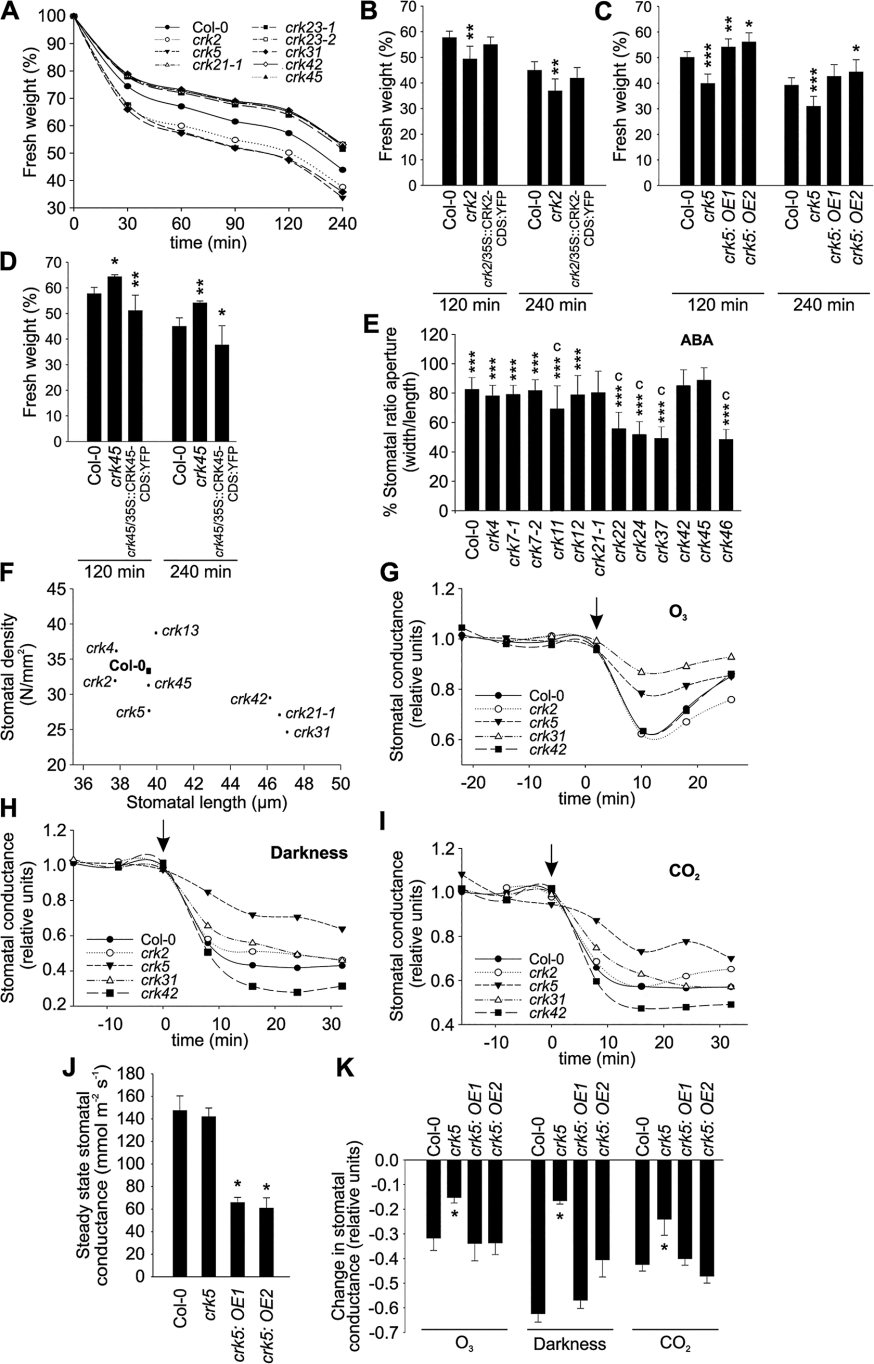
Stomatal development and responses are impaired in specific *crks*. **(A)** A subset of the *crk* mutants showed altered water loss (shown as decrease of fresh weight) compared to Col-0 wild type plants after detachment of shoots from roots as evaluated from rosette weight. Complementation of the *crk2* **(B)**, *crk5* **(C)** or *crk45* **(D)** mutants restored a wild type-like water loss phenotype as interpreted from decrease of fresh weight of excised rosettes. Asterisks indicate differences between *crk* mutants or complementation lines and Col-0 with statistical significance at *P<0.05, **P<0.01 and ***P<0.001 according to one-way ANOVA with *post hoc* Tukey HSD. The experiment was repeated three times with similar results. **(E)** Stomatal apertures were measured 2 h after abscisic acid (ABA) treatment. Some *crk* mutants are impaired in stomatal closure 2 h after treatment with 10 μM ABA. Results are means of % stomatal aperture ratio (width/length) ± SE (average number of stomata measured = 250). Asterisks indicate statistical significance between control and ABA treatment at *P<0.05, **P<0.01 and ***P<0.001 (linear model, single-step p-value adjustment). Lowercase letters indicate statistical significance between wild type Col-0 and *crk* mutant at *P*<0.05 (a), *P*<0.01 (b) and *P*<0.001 (c) according to one-way ANOVA with *post hoc* Dunnett’s test. **(F)** Stomatal density (number of stomata/mm^2^) is correlated with stomatal length (μm). Most of the *crks* exhibit a smaller stomata density which correlates with longer stomata (Pearson correlation -0.69, p-value = 0.04). Results are means (average number of stomata measured = 500). **(G-I)** Time courses of stomatal conductance (relative units) in response to a 3 min pulse of 500–600 ppb of O_3_ (**G**), darkness (**H**) and elevation of CO_2_ from 400 ppm to 800 ppm **(I)** in a subset of *crk* mutants and Col-0. Stimuli were applied at 0 time point, which is indicated by an arrow; pre-treatment stomatal conductance was used for normalization. Graph shows the mean of two experiments (*n* = 6). (**J**) Overexpression of CRK5 led to lower stomatal conductance compared to Col-0 wild type. (**K**) Complementation of the *crk5* mutant restored wild type-like phenotype in the response to a 3-min pulse of 500–600 ppb of O_3_, darkness, and elevating CO_2_ from 400 to 800 ppm. Asterisks indicate differences between *crk* mutants or complementation lines and Col-0 with statistical significance at *P<0.05 according to one-way ANOVA with *post hoc* Tukey HSD. The experiment was repeated three times with similar results.

Stomata are key structures in the control of plant responses to drought stress, pathogen infection, and other stimuli. Control of the stomatal aperture is a complex process involving plant hormones, most prominently ABA, ROS, and calcium (Ca^2+^) signalling to mediate and integrate plant-derived and environmental signals [34]. Transcriptional analysis suggested that several CRKs are involved in the control of drought responses (S7 Fig) [18]. Most CRKs displayed lower transcript abundance in guard cells compared to total leaf based on microarray meta-analysis (S14A Fig) while according to qPCR several *CRKs* displayed higher transcript levels in guard cells (S14B Fig). The difference between different methods to analyze gene expression could be due to the different preparation methods for guard cells. For the qPCR experiments enzymatic digestion was used for the isolation of guard cell protoplasts which might cause an additional pathogen treatment. As several *CRKs* have been described to show elevated transcript levels in response to pathogen or elicitor treatments, this could likely be the source of this difference. Water loss from detached leaves or rosettes can be used as a measure of initial stomatal aperture and the rate of stomatal closure. Water loss was enhanced in *crk2*, *crk5*, and *crk31*, as indicated by rapid decrease of rosette weight due to impaired stomatal regulation (Fig 5A and S4 Table). Complementation of the *crk2* and *crk5* mutations rescued water loss phenotypes of these mutants (Fig 5B and 5C). Several other *crks*, notably *crk45*, lost less water after detachment compared to Col-0 wild type (Fig 5A). Complementation of the *crk45* mutation using an overexpression construct (see materials and methods) rescued the phenotype and led to increased water loss compared to the mutant and Col-0 wild type (Fig 5D).

Stomatal openness (measured as the ratio of width to length) in response to the plant hormone ABA was altered in several *crk* mutants (Figs 5E and S15A), suggesting that CRKs may also participate in ABA-dependent control of the stomatal aperture. The *crk22*, *crk24*, *crk37*, and *crk46* mutants showed stronger ABA-induced stomatal closure compared to Col-0 wild type (Figs 5E and S15A). Similar to other mutants [35], we found a negative correlation between stomatal length and density for *crks* (Figs 5F and S15B). Furthermore, the analysis showed a cluster of *crks* that had reduced stomatal density and increased stomatal length as compared to Col-0 (S15B Fig).

**Fig 6:**
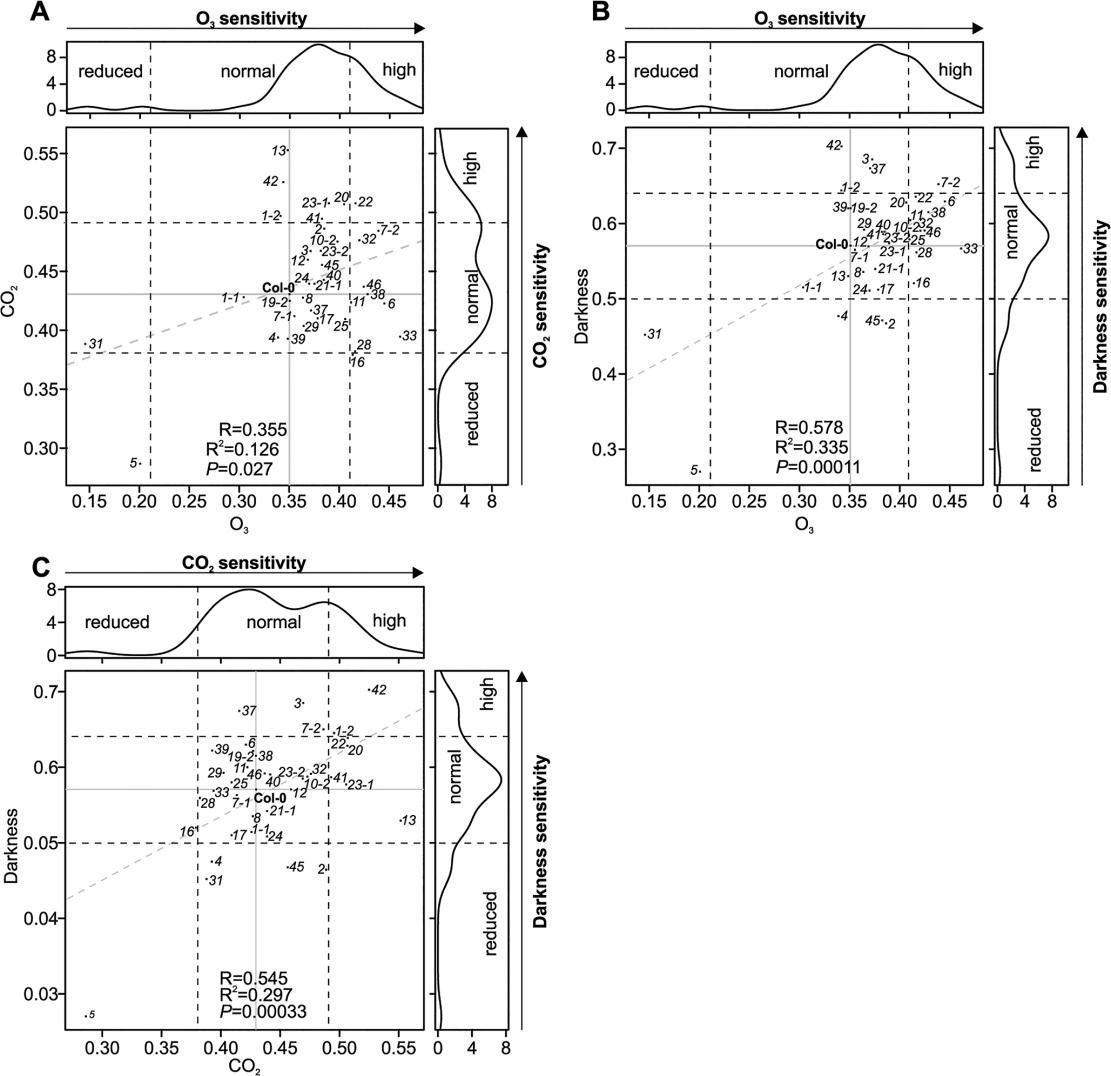
Scatter plots of stomatal regulation in *crk* mutants. The *crk5* was insensitive to all studied stimuli, whereas *crk31* was particularly insensitive to O_3_. The lines *crk19-1* and *crk22* were more sensitive to the analysed stimuli. **(A)** Scatter plot of stomatal responses of *crk* mutants to O_3_ (x-axis) and CO_2_ (y-axis). (**B**) Scatter plot of stomatal responses of *crk* mutants to O_3_ (x-axis) and darkness (y-axis) (**C**) Scatter plot of stomatal responses of *crk* mutants to CO_2_ (x-axis) and darkness (y-axis). Dashed lines indicate the cut-off for reduced, normal, and high response with respect to Col-0. Grey dashed lines show regression fit with correlation (R), coefficient of determination (R^2^) and significance reported in lower right corner in each plot. Reduced or increased responses were statistically significant in the majority of mutants (see respective barplots in S17, S18 and S19 Figs).

To compare microscopic measurements of the stomatal aperture with stomatal function we measured stomatal conductance in gas exchange experiments using intact, soil-grown rosettes [36]. Thirteen *crks* displayed slightly increased basal steady-state stomatal conductance under control conditions (S16A Fig). Under conditions that induce rapid stomatal closure [36] (elevated CO_2_, darkness, pulse of O_3_) the rapid decrease in stomatal conductance was less pronounced in *crk5* and *crk31* compared to wild type plants (Figs 5G–5I, S16B–S16D, S17, S18, S19 and S20). These two mutants also exhibited increased water loss (Fig 5A). Complementation of the *crk5* mutant restored wild type-like stomatal responses in the mutant in response to O_3_, darkness and CO_2_ (Fig 5J and 5K). Some *crk* mutants showed slightly increased stomatal closure compared to Col-0 wild type in response to the stimuli tested (S16, S17, S18, S19 and S20 Figs). In response to CO_2_, stomatal closure of *crk31* was also somewhat (but not statistically significantly) reduced (Figs 5I and S19). In addition to the major regulators, many additional CRKs may be involved in stomatal closure but are compensated by the redundancy within the gene family. This is suggested by the fact that the overall responses to O_3_, CO_2_, and darkness correlate significantly even when including the non-significant responses (Fig 6A–6C). This could result from compensation that is not perfect, as would be expected with genes which have slightly different structures (Fig 6A–6C).

CRKs have not been implicated in the regulation of stomatal openness and closure previously. Our findings suggest that specific CRKs are involved in controlling basal stomatal aperture and stomatal responses to environmental stimuli, which are critical to plant survival. In addition, CRKs also participate in the regulation of stomatal numbers.

### CRKs are integral components of plant defence

**Fig 7:**
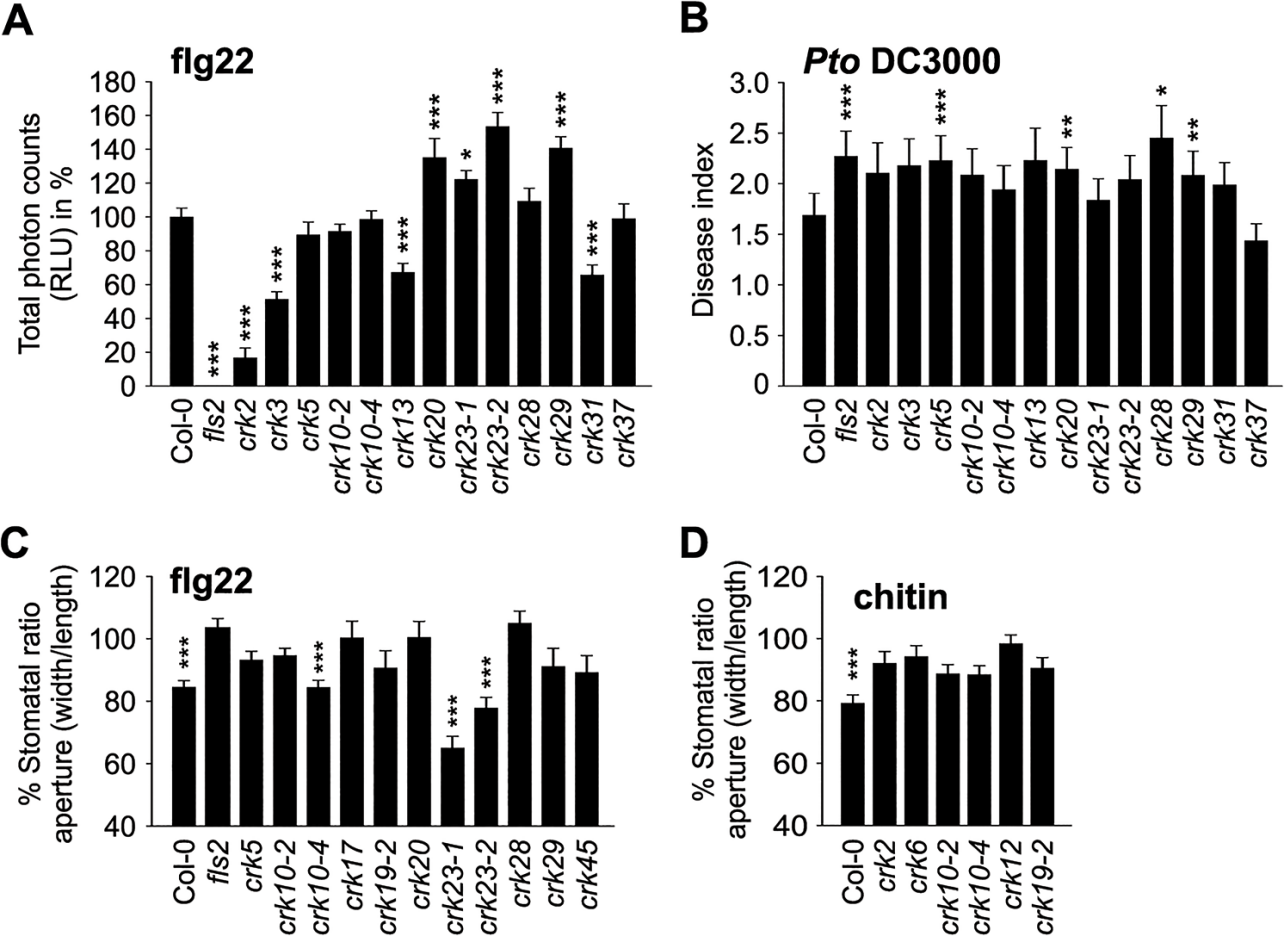
Immunity to bacterial pathogens is impaired in *crk* mutants. **(A)** ROS production was enhanced in several *crks* compared to Col-0 wild type after elicitation with 100 nM flagellin (flg22) in 4 week-old leaves. Data show the percentage of the mean of the total RLU (relative light units) to Col-0 ± SE (*n* = 24). Asterisks indicate differences between *crks* and Col-0, statistical significance *P<0.05, **P<0.01 and ***P<0.001 (one-way ANOVA *post hoc* Dunnett). **(B)** A subset of *crks* was more susceptible to *Pto* DC3000 (spray infection of 2-week old seedlings at 10^8^ cfu ml^-1^) compared to Col-0. Disease symptoms were scored 3 days post inoculation: 0, no symptom; 1, one symptomatic cotyledon; 2, two symptomatic cotyledons; 3, dead seedling. Results are means ± SE (*n* = 48). Asterisks indicate differences between *crks* and Col-0, statistical significance *P<0.05, **P<0.01 and ***P<0.001 (Mann-Whitney test, Benjamini-Hochberg correction for multiple comparisons). **(C)** Some *crk* mutants are impaired in stomatal closure after 2 h treatment with 10 mM flg22. Results are presented as mean of stomatal aperture ratio (width/length) after treatment compared to pre-treatment values in percentages ± SE (average number of stomata measured = 250). Asterisks indicate statistical significance between control treatment at *P<0.05, **P<0.01 and ***P<0.001 (linear model, single-step p-value adjustment). **(D)** Stomatal apertures were measured 2 h after chitin treatment. Stomatal closure is impaired in several *crk* mutants after treatment with chitin (1 g l^-1^) for two hours (five selected mutants are shown). Results are means of % stomatal aperture ratio (width/length) after treatment ± SE (average number of stomata measured = 250). Asterisks indicate statistical significance between control treatment at *P<0.05, **P<0.01 and ***P<0.001 (linear model, single-step p-value adjustment). All experiments were repeated three times with similar results.

Apoplastic ROS production also plays an important role in pathogen defence [37]. It can be triggered by treatments with pathogen-, microbe-, or damage-associated molecular patterns (PAMPs; MAMPs; DAMPs, respectively), for example flg22, a peptide derived from flagellin, an integral component of the bacterial flagellum [38,39]. Basal ROS production was slightly reduced in thirteen and elevated in two *crk* lines (S21 Fig). In response to elicitation with flg22 eleven *crk* lines displayed significantly increased ROS production while ROS production was decreased in *crk2*, *crk3*, *crk13*, and *crk31* (Figs 7A and S22A). This suggests that CRKs might be involved in control of ROS production, though the mechanism is not clear.

Several *crk* lines were more susceptible than the wild type to surface infection with the hemi-biotrophic bacterial pathogen *Pseudomonas syringae* pv. *tomato* DC3000 (*Pto* DC3000; Figs 7B and S22B). Notably, elevated flg22-induced ROS production did not fully match the responses to *Pto* DC3000 infection (Figs 7A and 7B and S22). The *crk5* and *crk28* mutants exhibited normal ROS production (Fig 7A) but showed increased disease symptoms (Fig 7B), while *crk23* showed elevated flg22-induced ROS production but did not differ from Col-0 wild type in its susceptibility to *Pto* DC3000. The *crk20* and *crk29* mutants were also more susceptible to infection by *Pto* DC3000 in spite of the elevated ROS production.

**Fig 8:**
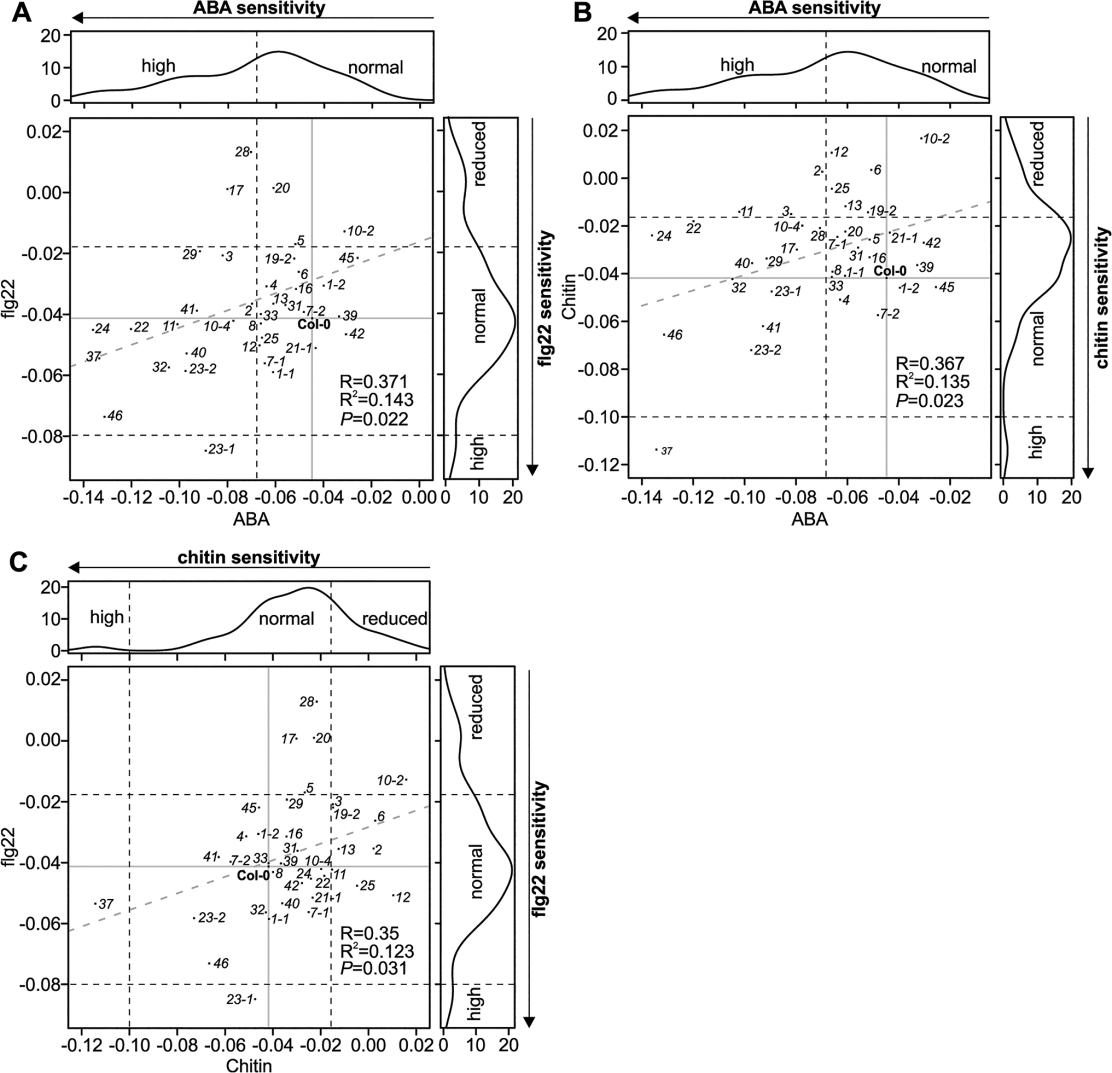
Scatter plots for stomatal regulation in *crk* mutants. **(A)** Scatter plot of stomatal responses of *crk* mutants to ABA (x-axis) and flagellin (flg22; y-axis). **(B)** Scatter plot of stomatal responses of *crk* mutants to ABA (x-axis) and chitin (y-axis). **(C)** Scatter plot of stomatal responses of *crk* mutants to chitin (x-axis) and flg22 (y-axis). Black dashed lines indicate the cut-off for reduced, normal, and high response with respect to Col-0. Grey dashed lines show regression fit with correlation (R), coefficient of determination (R^2^) and significance reported in lower right corner in each plot. Reduced or increased responses were statistically significant in the majority of mutants (see respective barplots in S15A and S23 Figs).

The different level of ROS production in the *crk* mutants compared to Col-0 wild type after elicitation with flg22 suggests that at least some CRKs might act through ROS signalling pathways rather than direct pathogen perception. To test this hypothesis, we measured PAMP-induced stomatal closure in Col-0 wild type and *crk* plants. PAMP perception through RLKs leads to NADPH oxidase activation and extracellular ROS production and induces stomatal closure [40–42]. While ROS production was normal or even elevated in most *crks*, stomatal closure triggered by flg22 was impaired in several mutants including *crk5*, *crk17*, *crk20*, and *crk28* (Figs 7C and S23A) and corresponds to their increased susceptibility to *Pto* DC3000 (Figs 7B and S22B). In response to the PAMP chitin, a fungal cell wall component, stomatal closure was reduced in several *crk* mutants including *crk2*, *crk6*, *crk10-2*, *crk10-4*, *crk12*, and *crk19-2* (Figs 7D and S23B). Significant ABA-, flg22-, and chitin-induced stomatal responses were mostly mediated by different CRKs (Fig 8A and 8B); only a few CRKs participated in more than one process. Responses to the PAMPs flg22 and chitin might be mediated mostly by different CRKs (Fig 8C). Again, in addition to the major regulators, the responses overall show a significant correlation even when including also the non-significant *crk* mutants (Fig 8A–8C). This may be due to the redundancy within the gene family, similar to the result observed in stomatal conductance (Fig 6A–6C). Most *crk* mutants that were affected in stomatal immunity displayed higher transcript abundance in guard cell protoplasts compared to whole, untreated leaves (S14B Fig).

**Fig 9:**
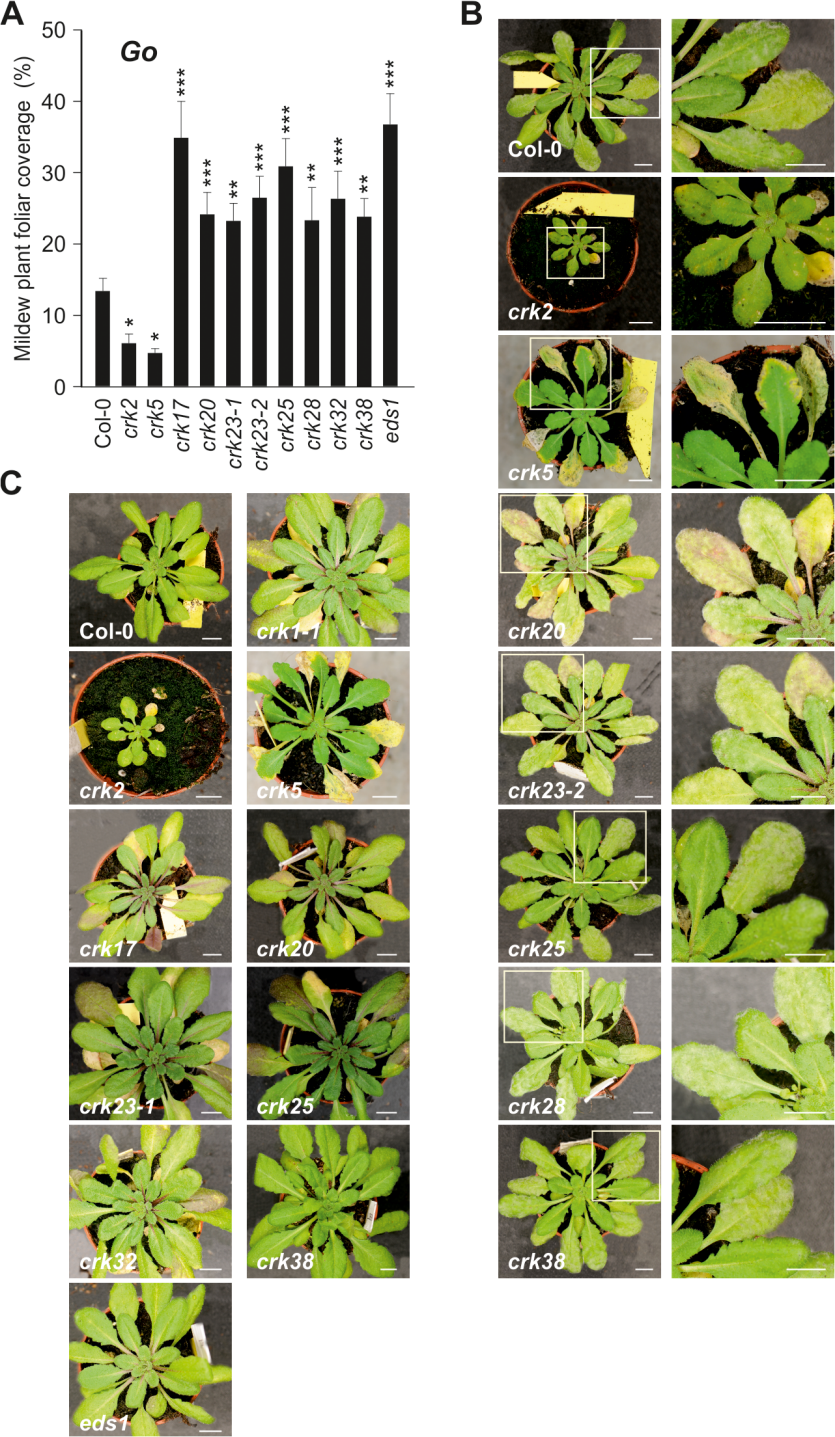
Immunity to powdery mildews is impaired in *crk* mutants. **(A)** Relative amount of plant foliar mildew coverage (in percent) caused by the virulent biotrophic powdery mildew *Golovinomyces orontii* (*Go*) on *crks* compared to Col-0 wild type and *Go-*super-susceptible *eds1*. Results are mean ± SE (*n* = 15). The experiment was conducted three times and the amount of disease was normalised between experiments by setting the infection cover of Col-0 to one. Asterisks indicate differences between *crks* and Col-0, statistical significance *P<0.05, **P<0.01 and ***P<0.001 (linear mixed model with Benjamini-Hochberg false discovery rate correction). **(B)** Pictures of *Go* infected *crks* and close-up of infected leaves. In some cases, infected leaves displayed increased pigmentation and *crk5* showed accelerated death of the infected leaves. Bar = 1 cm. **(C)** No infection was observed with the non-host powdery mildew *Blumeria graminis* f. sp. *hordei* (*Bgh*) but several *crks* displayed increased pigmentation. Bar = 1 cm. All experiments were repeated three times with similar results.

While *Pto* DC3000 can infect plant leaves through stomata [43–45], powdery mildews follow a different strategy by penetrating and colonizing epidermal cells [46–48]. Infection of wild type Col-0 with the biotrophic virulent powdery mildew fungus, *Golovinomyces orontii* (*Go*), or the non-host powdery mildew fungus *Blumeria graminis* f. sp. *hordei* (*Bgh*) resulted in altered expression of several *CRK* genes (S24A Fig) and susceptibility towards the pathogens was also affected in *crks*. Several *crks* showed increased susceptibility to *Go*, whereas responses to *Bgh* were more subtle. Specifically, *crk2* and *crk5* displayed less visible mildew symptoms in response to *Go* infection (Fig 9A and 9B), whereas *crk17*, *crk20*, *crk23-1*, *crk23-2*, *crk25*, *crk28*, *crk32*, and *crk38* were more susceptible (Figs 9A and 9B and S24B and S24C). Furthermore, *crk20* and both alleles of *crk23* (*crk23-1* and *crk23-2*) showed consistently enhanced pigmentation of the leaves following *Go* infection (Fig 9B). Additionally, *crk1-1*, *crk17*, *crk25*, and *crk32* showed this response also to the non-pathogenic *Bgh* (Fig 9C).

While previous reports have already suggested that CRKs participate in defence against bacterial pathogens, our results show that CRKs are also involved in the defence against fungal pathogens. CRKs participate in the control of pathogen-induced ROS signalling, stomatal responses and pre-invasive immunity.

## Discussion

**Fig 10:**
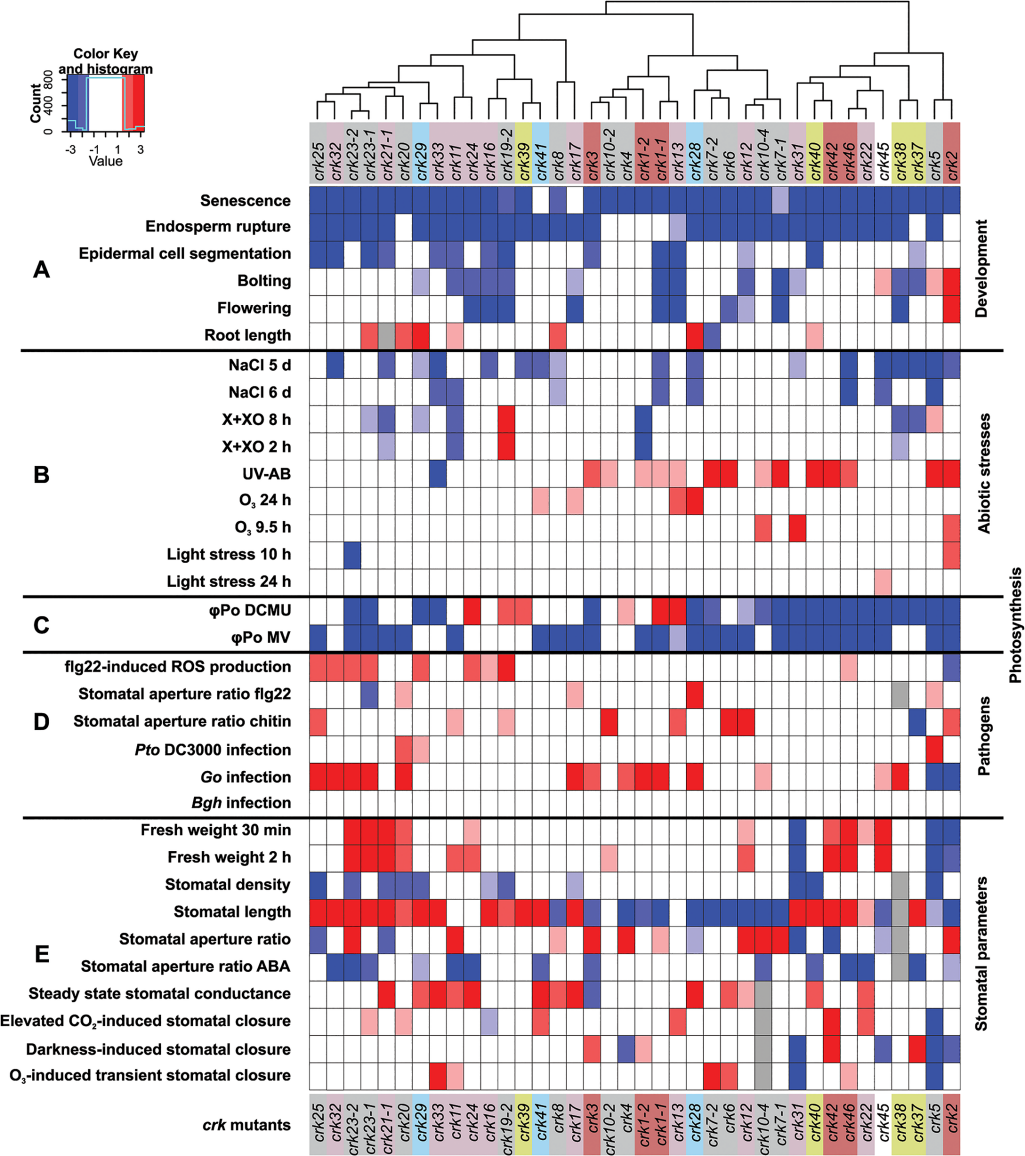
Integrated cluster analysis of *crk* mutant phenotypes. An age-matched collection of T-DNA insertion lines in *CRK* genes was analyzed for developmental and stress-related phenotypes. **(A)** Analysis of developmental phenotypes of *crk* mutant lines: senescence, germination (endosperm rupture), epidermal cell segmentation, bolting, flowering, and root length. **(B)** Analysis of abiotic stresses phenotypes of *crk* lines: germination of *crk* lines on medium containing NaCl, cell death (measured by electrolyte leakage) in response to Xanthine-Xanthine Oxidase (X+XO), ultraviolet light (UV-AB), ozone (O_3_), or light stress. **(C)** Analysis of photosynthesis responses upon treatment with DCMU or methyl viologen (MV). **(D)** Pathogen phenotypes. ROS production in response to treatment with the bacterial elicitor flagellin (flg22). Stomatal aperture ratio in response to flg22 and chitin treatments and *crk* susceptibility to the hemibiotrophic bacterial pathogen *Pseudomonas syringae* pv. *tomato* DC3000 (*Pto* infection) or the biotrophic fungal pathogens *Golovinomyces orontii* (*Go*) (virulent on *Arabidopsis*) or *Blumeria graminis* f.sp. *hordei* (*Bgh*, a barley pathogen, non-pathogenic on *Arabidopsis*). **(E)** Analysis of stomatal parameters: fresh weight (for determination of water loss), density, length, aperture, stomatal aperture in response to ABA treatment, steady state stomatal conductance, stomatal closure in response to elevated CO_2_, O_3_, and darkness. Experiments were made comparable by bootstrap sampling to *n* = 15 followed by averaging over bootstrap estimates. Red and blue indicate statistically significant increase or decrease in response compared to Col-0 wild type, respectively, while white indicates a response that is similar to wild type Col-0. The intensity of color is proportional to the Benjamini-Hochberg false discovery rate (FDR) adjusted Z statistic which takes the estimated means and their variation into account. As a rough guideline, |Z|>1.67 corresponds to a FDR<10% (shown with light hue), and |Z|<2.6 to a strong FDR<1% (intense color). White: non-significant response; grey: not measured. A corresponding plot displaying the adjusted Z statistics without thresholding is shown in S25 Fig.

This study addresses the physiological and cellular roles of the CRK protein family—with 44 members, one of the largest subgroups of RLKs in *Arabidopsis* (Fig 2). As a result of the large-scale phenotyping of a *crk* T-DNA insertion mutant collection we were able to identify clear and specific phenotypes for several *crk* mutants. Clustering of all phenotypic differences of *crk* mutants compared to Col-0 wild type allowed the generation of a genetic and phenotypic framework (Figs 10 and S25). Previous reports have linked ectopic overexpression of individual CRKs with pathogen defence and regulation of cell death [12,13,20] and CRK45, one of the few CRKs lacking ecto- and transmembrane domains, was found to interact with pathogen effectors in a large-scale screen [49]. However, it is unclear whether other CRKs could be direct targets for pathogen effectors. Meta-analysis of microarray data (S7 Fig) suggested the involvement of CRKs in response to a variety of additional stimuli beyond pathogen defence, including O_3_, UV, light, salt, and drought stress. Several *crks* displayed phenotypes in response to those stimuli (Figs 10 and S25). In addition, growth and development were altered in several *crks* (Figs 10 and S25).

Roles for ROS/redox signalling in biotic or abiotic stress response have been shown repeatedly over the last decade [20,50]. Similarly, plant development [51], root growth [52,53], senescence [54], germination [55], cell expansion [56], flowering [57–59], and cell cycle control [60] are tightly integrated with ROS and redox-dependent processes. This might suggest that CRKs could be connected to ROS/redox signalling in both stress and developmental processes. Three CRKs (CRK27, CRK34, and CRK44) might fulfil critical roles for plant survival. Their expression in different plant tissues and organs did not reveal any striking patterns (S5 Table) and no assumption towards their function. In the Col-0 genetic background no T-DNA insertion lines were obtained for CRK27, CRK34, and CRK44. Their roles will however need to be verified in the future.

Overall, some of the most striking phenotypes were found for *crk2* and *crk5*, members of the basal group I and group V, respectively (Fig 10). Some phenotypes of *crk2* might be caused by its dwarf morphology. However, this, together with the underlying cause of the dwarfism in *crk2*, will require more detailed analysis in the future.

Earlier studies have suggested that the high degree of amino acid sequence similarity between CRK family members would be the main reason for redundancy and lack of loss-of-function mutant phenotypes [13,14,21,22]. Our findings suggest that CRKs do not function in an exclusively redundant fashion. Specific CRKs, for example CRK2 of the basal phylogenetic group I, could function as primary regulators while others might provide calibration for more fine-tuned responses. This would offer an explanation for the intriguingly large number of CRKs in *Arabidopsis thaliana*, and also in other species.

Tissue and cell specificity of various *CRKs* might also be a reason for the large number of different genes, as they would be under different transcriptional regulation. However, according to eFP browser (http://bar.utoronto.ca/efp/cgi-bin/efpWeb.cgi) [61] most *CRKs* seem to be present in low levels in most tissues (S5 Table). Only *CRK2* and *CRK3* show strikingly higher transcript abundance in guard cells, hypocotyl and in vascular tissues (S5 Table). Expression of *CRKs* might however also be regulated in response to external stimuli. This is the case for *CRK7* [27] and *CRK5* [28].

**Fig 11:**
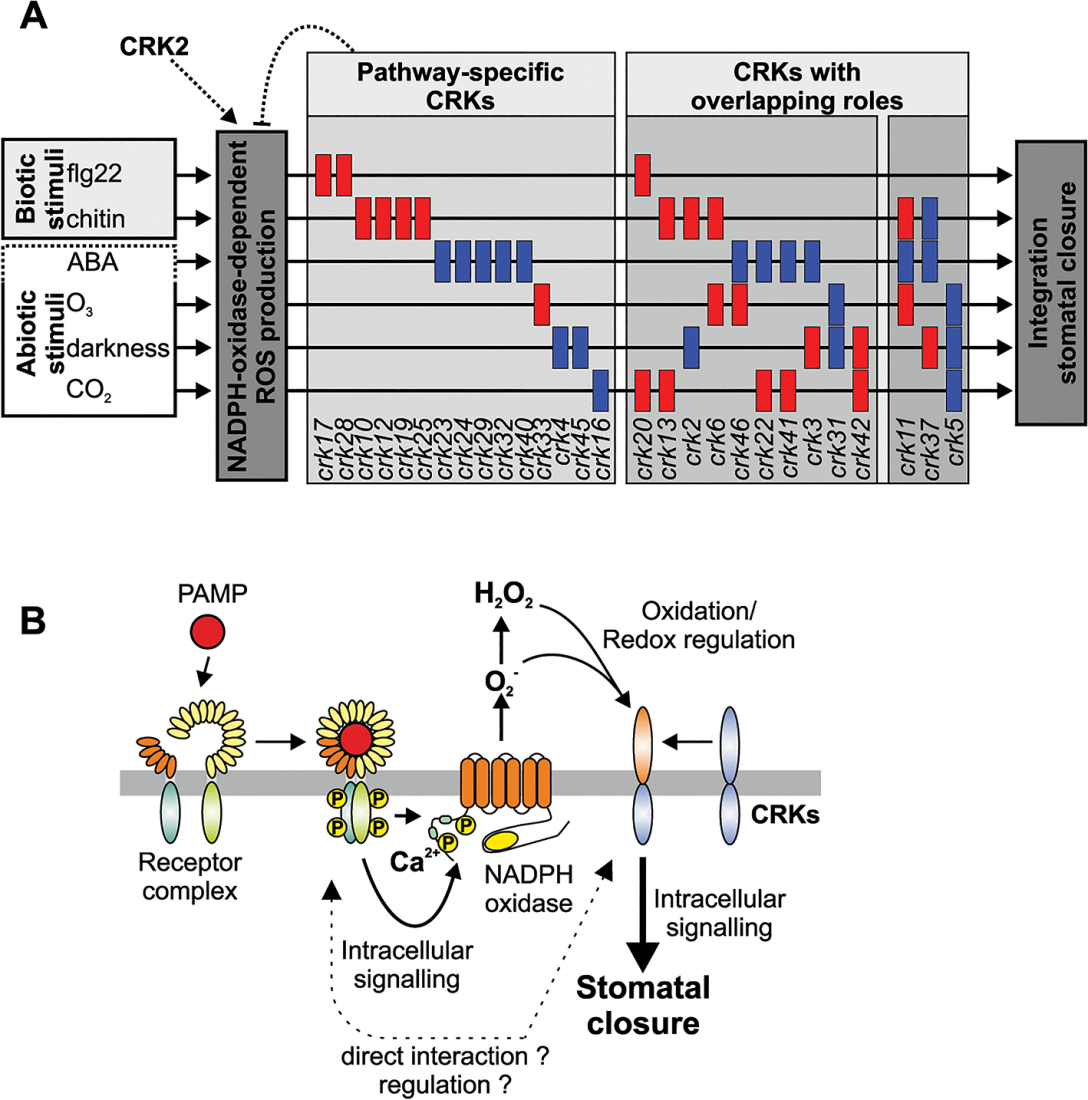
Models of CRK function and how they could provide specificity of stomatal aperture regulation. **(A)** CRKs might act as pathway-specific or multi-pathway regulators of stomatal aperture in response to the PAMPs flg22 and chitin but also the stress hormone ABA and the abiotic stimuli O_3_, darkness and CO_2_. The figure has been created from data presented in Fig 10. **(B)** CRKs are involved in the response to pathogens downstream of extracellular ROS production. PAMPs are recognized by pattern recognition receptor complexes. Subsequently, intracellular signalling leads to activation of extracellular superoxide production by NADPH oxidases. ROS perception subsequently leads to intracellular signalling and ultimately stomatal closure. CRKs are implicated in linking extracellular ROS production to intracellular signalling and might regulate and/or interact directly with the recognition receptor complexes.

Regulation of the stomatal aperture is an important factor in the response to a wide range of stimuli [45,62,63] and has been shown to involve ROS signalling [64]. Several *crks* showed differences in ABA- or stress-induced stomatal closure (Fig 11A). Thus, it is tempting to link the *crk* phenotypes defective in ABA signalling with altered ROS signalling. Two *crks* (*crk5* and *crk31*) showed defective responses to stomatal closure induced by abiotic factors. Expression of *CRK5* is not restricted to guard cells (S14B Fig), underlining the importance of whole-leaf processes mediated by CRK5 in stomatal movements. CRKs might also be involved in determining basal openness of stomata. Interestingly, different CRKs control basal steady-state stomatal openness and stomatal responsiveness to stimuli.

Stomatal closure can also be triggered by application of PAMPs, *e*.*g*. flg22 or chitin. Signal transduction from receptor-mediated PAMP perception by intracellular signalling and ROS production down to stomatal closure is becoming increasingly better understood [45,65]. There are convergence points in the signalling pathways of ABA- and PAMP-induced stomatal closure, for example ROS production by the NADPH oxidase RBOHD [34,41,66]. Consistent with enhanced disease symptoms upon *Pto* DC3000 infection of several *crks*, mutations in several CRKs impaired flg22-triggered stomatal immunity (Fig 11A). The results suggest that PAMP perception and the earliest signalling events upstream of ROS production are likely not affected in *crk* mutants. Chitin-induced stomatal closure was also compromised in *crk*s, but different *crks* were affected in chitin-induced stomatal closure compared to flg22 (Fig 11A) suggesting that CRKs could provide signalling specificity.

Even though powdery mildew fungi do not use stomata as an infection route but directly penetrate epidermal cells, several *crks* impaired in chitin-induced stomatal immunity also displayed enhanced susceptibility to *Go*. This indicates that CRKs are also signalling components in guard cell function-independent plant defence responses. Interestingly, *crk* mutants that were impaired in stomatal immunity were not altered in ABA-induced stomatal closure (Fig 11A). Together, this indicates that many CRKs might fulfil independent functions in PAMP- or ABA-triggered processes in guard cells (Fig 11A), while a few CRKs might control common, and presumably basal, aspects of guard cell function (Fig 11B). While CRKs in *Arabidopsis thaliana* are involved in immune signalling evidence from legumes suggests that CRKs might also participate in the control of symbiosis or even in distinguishing between pathogenic or beneficial microbes [22]. This suggests that there might be additional functions for CRKs which cannot be addressed in *Arabidopsis*.

The most prominent feature of the CRK protein family is the presence of two cysteine-rich DUF26 domains (with C-X_8_-C-X_2_-C-motifs) in the extracellular region. Structural analysis of the DUF26 domain of ginkbilobin-2 (Gnk2), a *Ginkgo biloba* protein containing a single DUF26 domain with a proposed function as an antimicrobial protein, suggests that the cysteines form disulphide bonds [67,68]. The role of the CRK ectodomain is still unknown. It could either bind a ligand (peptide or other) or be crucial for the formation of complexes with other receptors. Recently, it has been suggested that the DUF26 domain in Gnk2 might be involved in mannose binding [69]. The residues required for mannose binding in Gnk2 are, however, not conserved in the DUF26 domains of the CRKs. It has also been suggested that the cysteines in the DUF26 domain could be a target for redox modification which might lead to a conformational change, for example through opening of disulphide bridges. However, redox regulation of CRK ectodomain structure and ligand binding might not be mutually exclusive.

A connection between CRK function and redox or ROS-related processes is also suggested by the strict and specific regulation of genes encoding CRKs under ROS-producing conditions [7,19]. Through this CRKs might participate in feedback regulation of ROS production where they might sense extracellular ROS and be part of a “ROS amplification loop”. This would place the CRKs in the “ROS wave” [70] by perceiving ROS from neighbouring cells and transducing the signal into the cytosol, subsequently regulating NADPH oxidase activity and signal propagation (Fig 11B). This is particularly interesting since the precise control and adjustment of ROS production in response to different stimuli is still unresolved even though activation and regulation of NADPH oxidases and other ROS-producing enzymes through protein phosphorylation and RLKs is becoming better understood. Proteins with DUF26 domains are restricted to plants but other cysteine-rich domains could fulfil analogous functions to CRKs with respect to sensing of extracellular ROS in other organisms. The data shown here suggests that one of the main functions of CRKs could be to provide signalling specificity downstream of extracellular ROS production. However, it is unclear how this regulation might work exactly. Recent evidence suggests that CRKs might be able to interact with pattern recognition receptors [22] but the specificity and the precise role of this interaction will require further investigation. It suggests however, that CRKs might act in concert with other receptors and RLKs, possibly also during the regulation of plant development and during abiotic stress responses.

From the phenotypic framework (Figs 2 and 10) it will now be possible to dissect the molecular mechanisms through which the CRKs function. Conceptually, perception of cell-to-cell or environmental signals through CRKs could follow different modes of action as positive or negative regulators. However, it is likely that CRKs have multiple rather than single downstream targets. How CRK signalling is integrated in synergistic or antagonistic fashion might be highly process specific. The genetic and phenotypic framework and the proposed models for modes of CRK action will allow targeted and detailed mechanistic analysis of CRK function in the future. Ultimately, this will allow improvement of plant growth and tolerance to complex environmental challenges. Our results demonstrate that discovery of subtle phenotypic responses and aspects, which might otherwise be missed, can be facilitated with thorough phenotypic analysis of comprehensive mutant collections for large gene families, instead of studying individual family members.

## Materials and Methods

### Plant materials

All T-DNA insertion *crk* lines were obtained from the Nottingham *Arabidopsis* Stock Centre (NASC, http://nasc.life.nott.ac.uk/) and were confirmed by PCR (primers are listed in S1 Table). An age-matched seed collection was generated and used for all experiments. The seed collection has been donated to the European Arabidopsis Stock Centre in Nottingham (http://www.arabidopsis.info) and can be obtained from there.

### Growth conditions

Siliques were harvested at maturity and dried at room temperature for 10 days prior to the collection of seeds. Freshly harvested seeds were after-ripened for 3 months at 20°C (approximately 30% relative humidity) in darkness and further used in germination tests.

After stratification for 3 days at 4°C, seeds were grown in a mixture of soil and perlite (3:1) or on Jiffy Peat Pellets in the growing room under the following conditions: 8/16 h photoperiod, temperature 22/18°C (day/night, respectively), relative humidity of 70 ± 5%, and PAR (100–150 μmol m^-2^ s^-1^). Experiments were performed on 4 week-old plants, unless otherwise stated. For pathogen assays and stomatal analysis *Arabidopsis thaliana* plants were grown on general soil (*Arabidopsis* mix, John Innes Centre, Norwich), or for infection assays on Jiffy pellets (Jiffy Products, Norway) under 10 h or 16 h of light at 20–22°C and 65% humidity. Mutant *fls2* lines have been described previously [71].

### O_3_ and light stress experiments

O_3_ exposure and high-light treatments started at 9 am and were continued for 6 h. 18-day-old plants were used for O_3_ experiments. Leaf rosettes were harvested 7 h after the start of the treatment then washed with ultra-pure water and transferred into 15 ml ultra-pure water for electrolyte leakage measurements (*n* = 4). Experiments were repeated twice.

Ozone (350 ppb) and high-light (1430 μmol m^-2^s^-1^ photosynthetically available radiation [PAR; 400–700 nm], 11 mW m^-2^ UV-B radiation [280–315 nm], 25.4 W m^-2^ UV-A radiation [315–400 nm]) treatments were performed at the Research Unit Environmental Simulation of the Helmholtz Zentrum München (Germany) in the walk-in-size chambers and in a small sun simulator respectively. Spectral measurements were performed using a double monochromator system TDM300 (Bentham, Reading, England). *Arabidopsis* plants were cultivated on multiplication substrate (Floradur) mixed with quartz sand (Dorfner) in the respective ratio 5:1. After a 2-day pretreatment at 4°C, pots were cultivated under the following conditions: 250 μmol m^-2^ s^-1^ PAR under the exclusion of UV radiation (<400 nm), under 12h-day length (day: 23°C, 70% relative humidity; night: 18°C, 90% relative humidity).

### Xanthine-xanthine oxidase experiments

Extracellular superoxide was generated by vacuum infiltration of 1 mM xanthine (X) and 0.1 U ml^-1^ xanthine oxidase (XO, Sigma-Aldrich) into the leaf discs from 4-week-old plants as previously described [33,72]. Cell death was monitored by electrolyte leakage measurements with a conductivity meter (Mettler Toledo) at the indicated times after the end of the treatment.

### Confocal microscopy and image analysis

High-throughput confocal imaging was performed using the Opera microscope (PerkinElmer, Germany) as published [73]. For quantification of cell numbers, cotyledons were stained with propidium iodide according to Lucas *et al*. [74] and measured using PDQUANT as described previously [73].

### Pathogen inoculation

Bacterial inoculation assays were performed as described previously [75]. Briefly, *Pto* DC3000 was sprayed onto leaf surface at 10^8^ CFU ml^-1^ and disease symptoms were scored 3 days post inoculation.

For mildew infection, plants were grown under 8/16 h photoperiod (200 μmol m^-2^ s^-1^) at 22°C ± 1°C. Three to four week old plants were inoculated in a settling tower with about 1 spore per mm^2^ of a virulent *Golovinomyces orontii* (*Go*) powdery mildew isolate or 10 spores per mm^2^ of a non-host *Blumeria graminis* f. sp. *hordei* (*Bgh*) powdery mildew isolate. Symptoms and mildew coverage was assessed after 6 days. Mildew coverage in percent per plant was scored from digital images using the image processing software ImageJ (http://imagej.nih.gov/ij/). The experiment was conducted three times with 5 replicate plants.

### Quantitative real-time reverse transcription polymerase chain reaction (qPCR) analysis

Plants for qPCR were grown as above, samples were taken at 6, 16, and 24 h after inoculation with *Go* or *Bgh*. Five plants were pooled into one sample and the experiment was conducted three times. RNA extraction and cDNA synthesis was as described earlier [46] (primer sequences for *CRK* transcripts [7]). Relative *CRK* gene expression was analyzed by the comparative CT method. CT values were normalized to 18S rRNA and expression between uninfected control and *Go*/*Bgh* powdery mildew infected plants were compared using the 2-ΔΔCt method. Significant differences were determined according to student’s t test.

Guard cells were isolated as described [76] and cDNA was generated from RNA isolated from guard cell protoplasts. Expression of CRKs was compared with cDNA from total RNA isolated from untreated *Arabidopsis* leaves. *Actin-2* (At3g18780), *YLS8* (At5g08290), *PP2A* (At1g13320), *TIP41* (At4g34270), and At4g35510 were used as normalization genes for the analysis using the 2-ΔΔCt method.

### Bioassays for PAMP-induced responses

ROS assays were performed as described previously [41]. Briefly, 16 leaf discs were excised per genotype of four weeks-old plants and treated with 1 μM flg22. ROS was measured with a Varioskan multiplate reader (Thermo Fisher Scientific, USA) for 35 min.

### Electrolyte leakage measurement after UV-A and-B treatment

For ultraviolet light source, UVC 500 Crosslinker (Hoefer Pharmacia Biotech, San Francisco, CA, USA) equipped with three UV-B lamps (type G8T5E, Sankyo Denki, peak wavelength 306 nm) and two UV-A lamps (type TL8WBLB, Philips, peak wavelength 365 nm) were used. Plants were exposed to single radiation episode until a cumulative dose of 1500 mJ cm^-2^ was reached (roughly 10 minutes). After 4 days leaves were excised, fresh weight measured (g) and transferred into 50 ml falcon tubes containing 35 ml MilliQ water. The relative electrolyte leakage was measured with a conductance meter (WTW, INOLAB Cond Level 1) and calculated as a ratio between the value obtained after 1 h incubation and the total electrolyte leakage evaluated after autoclaving the samples.

### Analysis of the fluorescence transients (O-J-I-P test) upon MV and DCMU treatment

The O-J-I-P test was performed as described [77] using FluorCam and the associated software (Photon System Instruments, Czech Republic). Plants were dark-adapted for 30 min prior to measurement. The maximum quantum yield of primary photochemistry (*φ*_Po_), the size of the plastoquinone pool (qPQ) and the total dissipation of untrapped excitation energy from photosystem II (PSII) reaction center (DLo/RC) were calculated. *φ*_Po_ represents the probability that an absorbed photon is trapped by the reaction center and used for primary photochemistry, reducing Q_A_ to Q_A_^-^. Analysis of the fluorescence transients was made on whole rosettes and included two sets of plants: one set sprayed with 32 μM MV and kept in 8/16 photoperiod for two days and a second set sprayed with 20 μM DCMU (dissolved in 75% ethanol) and kept in darkness for two hours.

### Endosperm rupture assays

To assess germination of *crk* mutants, endosperm rupture assays were performed by placing after-ripened seeds in 9 cm Petri dishes (30 seeds per dish, three independent biological replicates) on 1% agar with addition of 0.01% PPM (Plant Preservative Mixture, Plant Cell Technology, USA). All assays were performed at 20°C under PAR of 200 μmol m^-2^ s^-1^. Testa and endosperm rupture were assessed every 5 hours up to 51 hours of imbibition. A seed was considered as germinated when the radicle protruded through both envelopes.

### Root length, bolting, flowering and senescence assays

Seeds were sterilized, germinated and grown as described [78]. Root length measurements were performed 8 days after stratification in two independent assays with 6 plants each. Interesting lines were additionally screened twice.

To measure bolting, flowering and senescence germinated seeds were transferred to soil and grown with 16 h-light/8 h-dark photoperiods at 22°C. Plants were considered bolting at the first appearance of the inflorescence, flowering at the opening of the flower petals and senescing at the first yellowing of the rosette leaves.

### Salt stress

Salt treatment assays were performed by placing seeds in Petri dishes (15 seeds per lines, three independent biological replicates) on MS medium containing 1% sucrose, 1% agar, buffered to pH 5.7 with 2.8 mM MES, with or without 120 mM NaCl; under 12h-day length (100–150 μmol m^-2^ s^-1^, day: 22°C—night: 19°C, 70% relative humidity). A seed was considered as germinated when the 2 cotyledons were visible. For each line, germination rate was expressed as the ratio (germination percentage on NaCl plates /germination percentage on control plates), at 5 and 6 days after stratification.

### Fresh weight (for water loss analysis)

Plants were grown as described in the plant materials section. Weight of detached whole rosettes was followed until 4 h at room temperature (five 3-week old plants per lines, for three independent biological replicates).

### Whole plant gas exchange experiments

To analyse steady-state stomatal conductance and stomatal responses to darkness, elevated CO_2_ and O_3,_ 21–26 days old plants and a custom made gas exchange device were used. As *crk2* had reduced growth rate, older plants (26 to 32 days old) were analysed. The device and plant growth conditions have been described previously [36,79]. First, plants were inserted into the device and kept at 150 μmol m^-2^ s^-1^ light, 65% air humidity and ambient CO_2_ concentration (400 ppm) until stomatal conductance had stabilized. To address stomatal response to darkness, light was switched off, to address elevated CO_2_-induced stomatal closure, CO_2_ concentration was increased to 800 ppm and in order to address stomatal closure induced by O_3,_ a three min pulse of 500–600 ppb of O_3_ was applied. In all experiments, stomatal conductance was followed for 32 min from application of closure-inducing stimuli.

### Complementation lines

*CRK2* and *CRK45* coding regions were cloned into pDONRzeo (Invitrogen) *via* Gateway site-specific recombination. Coding regions were then assembled together with CaMV 35S-promoter and Venus YFP [80] C-terminal tag into the pBm43GW [81] expression vector, using MultiSite Gateway technology (Invitrogen), creating a translational fusion protein. Primers are listed in S1 Table. Constructs were transformed into the corresponding T-DNA insertion plants by GV3101 *Agrobacterium*-mediated floral dipping [82]. For selection of successful transformants, seeds were plated on ½ MS media supplemented with 1% sucrose and 20 μg/mL Basta. Plants were grown for one week *in vitro* before being transferred to soil. T1 plants were used for experiments with each plant constituting an individual insertion event.

*CRK5* complementation/over-expression lines have been described in Burdiak et al. [28]. Two individual homozygous T3 lines with a single insert were used.

### Data analysis

All data analysis was carried out using R, version 2.15.1.

### Estimation of Z scores

All experiments were made commeasurable by computing the Z score statistic of the comparison of each of the alleles versus the Col-0 wild type reference using appropriate statistical test for the given data set. Sample sizes across different experiments were normalised by bootstrap sampling such that in each bootstrap data set the number of samples for a given condition was set to *n* = 15. The Z score for each experiment was computed as the mean of the Z score estimates from bootstrap data sets. In cases where the null distribution of the statistical test did not follow Z distribution (*e*.*g*. with Mann-Whitney test), it was approximated with the Z-distribution. To reduce the number of false positives produced by the analysis, the Z-scores in each experiment were transformed to P-values and corrected using Benjamini-Hochberg false discovery rate adjustment. The adjusted P-values were then transformed back to obtain adjusted Z-scores. Adjusted Z-score values >2 or < -2 (corresponding to a false discovery rate < 5%) were considered to be statistically significant. In experiments involving a time course and many measurements, each time point was analysed separately to have statistical power comparable to non-time course experiments.

The list of specific statistical models and comparisons that were used to get Z scores and subsequently used for construction of heat maps are displayed in S6 Table. Unless stated otherwise, the Z score statistic from the statistical model was obtained comparing each *crk* genotype to Col-0 wild type.

Comparisons applying linear models were carried out with multcomp R package (http://cran.r-project.org/web/packages/multcomp/index.html). Heatmap of adjusted Z scores was constructed using hierarchical clustering with Ward’s method and applying Euclidean distance metric.

### Estimation of phylogenetic trees

Phylogenetic trees were estimated using MEGA6 [83]. Alignments were carried out using Muscle [84,85]. Trees for the entire coding region were estimated using all positions while trees for the kinase domains and extracellular regions were estimated using complete deletion for gaps. The initial guide tree was estimated using maximum parsimony. 1000 bootstrap replicates were used for all trees.

### Estimation of statistical significances

Statistical significances were estimated by constructing separate linear mixed model for each of the *crk* genotypes including Col-0 wild type as the reference data, modelling different time points as fixed effects. Comparisons were carried out using multcomp package for R (http://cran.r-project.org/web/packages/multcomp/index.html) and applying single step p-value adjustment for multiple comparisons. In light stress and fresh weight experiments, the model consisted of genotype, time and their interaction as fixed effects, and experiment replicate as random effect. Each *crk* genotype was compared to Col-0 in all time points. In O_3_, X+XO, salt experiments, the linear mixed model consisted of genotype, time and their interaction as fixed effects, and experiment replicate as random effect. Each *crk* genotype was compared to Col-0 under treatment in all time points.

In *Go* scoring, the leaf coverage percentage was modelled with genotype as fixed effect and experiment replicate as random effect. In germination assay analysis, the linear mixed model consisted of genotype, time and their interaction as fixed effects. In both experiments, each *crk* genotype was compared to Col-0.

### Gene expression data

Pre-processing of the gene expression data and the accession numbers in databases has been previously described [86]. In brief, the data was downloaded from NASCArrays (http://affymetrix.arabidopsis.info/narrays/experimentbrowse.pl), ArrayExpress (http://www.ebi.ac.uk/microarrayas/ae/), Gene Expression Omnibus (http://www.ncbi.nlm.nih.gov/geo/), and The Integrated Microarray Database System (http://ausubellab.mgh.harvard.edu/imds). Arrays were normalised with Robust Multi-array Average (RMA) [87], and log2 ratio of the mean of treatment and control expressions across biological replicates was computed, resulting in 141 differential expression profiles. Bayesian Hierarchical Clustering of CRKs present on the arrays was carried out using R package BHC [88] using log2 fold change ±1 as discretization threshold.

## Supporting Information

S1 Fig Phylogenetic clustering of the *Arabidopsis thaliana* CRK group of RLKs. **(A)** The intracellular part including the protein kinase domain of the CRKs was aligned using Muscle and the phylogeny was estimated with the Maximum-Likelihood method. **(B)** The ectodomain after the signal peptide to the start of the transmembrane domain was aligned using Muscle and the phylogeny was estimated using the Maximum-Likelihood method. CRK43 (At1g70740), CRK44 (At4g00960) and CRK45 (At4g11890) are lacking the signal peptide, the CRK ectodomain (ED) and transmembrane domain. The maximum-likelihood phylogenetic tree was estimated in MEGA6. The initial guide tree was constructed using maximum parsimony. Values at branch nodes represent bootstrap values (1000 replicates)
(EPS)

S2 Fig Position and orientation of the *CRK* genes in *Arabidopsis thaliana* chromosomes. The number determines the *Arabidopsis thaliana* chromosome number. Arrows indicate the orientation of genes. Colours are related to the five groups emerging from phylogeny (S1 Fig). The figure was realized using the Chromosome Map tool available at TAIR (http://www.arabidopsis.org/jsp/ChromosomeMap/tool.jsp).
(EPS)

S3 Fig Position of the T-DNA insertions in *crk* lines. *CRK* UTR regions, exons and introns have been drawn using the application Exon-Intron Graphic Maker (http://wormweb.org/exonintron). Open triangles indicate the position of the T-DNA insertion. Numbers indicate the different alleles used for the *crk* lines (*crk1-1/2*, *crk7-1/2*, *crk10-1/2/3/4*, *crk19-1/2*, *crk21-1/2*, *crk23-1/2*, and *crk43-1/2*). Arrows indicate primer positions used for RT-PCR. The putative pseudogene *CRK35* (At4g11500; DUF26 44) and the truncated *CRK9* (At4g23170) were excluded from this collection. No homozygous T-DNA insertion lines in Col-0 background were obtained for *CRK27*, *CRK34* and *CRK44*. Bar = 100 bp.
(TIF)

S4 Fig RT-PCR-based expression analysis of the T-DNA insertion *crk* lines. **(A)** Agarose gel electrophoresis of amplicons derived from RT-PCR experiments using total RNA from homozygous T-DNA insertion *crk* plants (*n* = 5). The upper and lower panels show amplicons produced using the corresponding *CRK* and *ACT2* gene specific primers respectively. No amplicons were obtained with *CRK1*, *CRK32*, *CRK33* and *CRK46* gene specific primers. Control reaction (ctrl) was performed without nucleic acid. **(B)** Table shows the number of PCR cycles used for the different T-DNA insertion *crk* lines, with the following PCR program: 1) 95°C—3 min; 2) 95°C—30 s; 3) 55°C- 30s, 4) 72°C -30 s, repeat x times from step 2 to 4. PCR with *ACT2* has been made with 22 cycles.
(EPS)

S5 Fig Representative pictures of 17-day old *crk* seedlings. Plants were grown under the following conditions: 250 μmol m^-2^ s^-1^ under 12 h-day length (day: 23°C, 70% relative humidity; night: 18°C, 90% relative humidity). Bar = 1 cm. Pictures are representative of three independent experiments.
(TIF)

S6 Fig Plant development is affected in several *crk* mutants. **(A)** Senescence was measured as days after flowering. Results are means ± SE (*n* = 8). **(B)** Flowering was measured as days after stratification. Results are means ± SE (*n* = 8). **(C)** Eleven *crk* mutants exhibit a lower pavement cell density (number of pavement cells/mm^2^) in 2 weeks old cotyledons. Results are means (*n* = 15) of detected cells using the CellQuant algorithm (ACapella software) in images captured by the OPERA high-throughput confocal imaging system. Asterisks indicate statistical significance at *P<0.05, **P<0.01 and ***P<0.001 (± SE, one-way ANOVA *post hoc* Dunnett) for (A, B, and C). All experiments were repeated three times with similar results.
(EPS)

S7 Fig Cluster analysis of *CRK* expression in response to biotic and abiotic stresses. Publicly available *Arabidopsis* microarray data was clustered to reveal patterns in transcriptional regulation of *CRKs* using Bayesian Hierarchical Clustering (BHC). Red and blue indicate increased or decreased expression compared to untreated plants, respectively. The intensity of the colors is proportional to the absolute value of the fold difference. Green boxes highlight regulatory clusters. Red dashed lines are cluster merges the BHC algorithm prefers not to make. The numbers on the branches are the log odds for merging the clusters.
(EPS)

S8 Fig Response of *crk* mutants to high light stress treatment. Electrolyte leakage was measured at different time points after initiation of the high light treatment (1450 μmol m^-2^ s^-1^, 6 h). Electrolyte leakage was measured from high light-treated (1450 μmol m^-2^ s^-1^, 6 h) 18-day old plants and expressed as percentage of total ion content of each plant (*n* = 4). Values represent means ± SE. Asterisks indicate differences between *crk* mutants and Col-0 with statistical significance at *P<0.05 (linear model with single-step p-value adjustment). The experiment was performed twice with similar results.
(EPS)

S9 Fig Chloroplastic ROS signaling is affected in *crk* mutants. The maximum quantum yield of primary photochemistry (φPo) in plants treated with 20 μM 3-(3,4-dichlorophenyl)-1,1-dimethylurea (DCMU) for two hours **(A)** or 32 μM methyl viologen (MV) for 48 hours **(B)**. φPo is calculated from the OJIP test as a ratio TR_0_/ABS, and represents the probability that an absorbed photon is trapped by the reaction center and used for primary photochemistry, reducing QA to QA^-^. Significant differences in relation to wild type are indicated (*n* = 12, bars represent SD) with statistical significance at *P<0.05, **P<0.01 and ***P<0.001 according to ANOVA using Tukey’s HSD *post hoc* test. Experiments were repeated three times with similar results.
(EPS)

S10 Fig Response of *crk* mutants to O_3_. Electrolyte leakage was measured at different time points after the start of O_3_ fumigation (350 ppb, 6h). Electrolyte leakage was measured from clean air (control) and O_3_-treated (350 ppb, 6 h) 18-day old plants and expressed as percentage of total ion content of each plant (*n* = 8). Results are means ± SE, asterisks indicate differences between *crk* mutants and Col-0 with statistical significance at *P<0.05, **P<0.01, ***P<0.001 (ANOVA using Tukey’s HSD *post hoc* test). The experiment was performed twice with similar results.
(EPS)

S11 Fig Images of *crk* mutant plants after exposure to O_3_. Representative pictures of O_3_-induced damage in *crk* seedlings observed at 32 h after initiation of an O_3_ fumigation (350 ppb, 6h). Lines were grown under the following conditions: 250 μmol m^-2^ s^-1^ under 12 h day length (day: 23°C, 70% relative humidity; night: 18°C, 90% relative humidity) during 17 days. On 18^th^ day, plants were exposed to O_3_ (350 ppb, during 6 h). Pictures were taken 32 h after the initiation of the O_3_ fumigation; pictures are representative of two independent experiments (*n* = 6). Bar = 1cm.
(TIF)

S12 Fig Response of *crk* mutants to extracellular superoxide production. Electrolyte leakage was measured after xanthine-xanthine oxidase treatment (0.1 U ml^-1^, 4 h). Electrolyte leakage was measured from xanthine (X, control) and xanthine + xanthine oxidase-infiltrated (X+XO, 0.1 U ml^-1^) 3 weeks old leaf discs and expressed as percentage of total ion content (*n* = 16). Values represent means ± SE and asterisks indicate differences between *crk* mutants and Col-0 with statistical significance at *P<0.05, **P<0.01 and ***P<0.001 (linear model with single-step p-value adjustment). The experiment was repeated three times with similar results.
(EPS)

S13 Fig Abiotic stress response in *crk* mutants. **(A)** Treatment with ultraviolet A and B radiation led to increased cell death in several *crks* compared to wild type as measured by relative electrolyte leakage. Mean values ± SD from three independent experiments are presented (*n* = 12). (**B** and **C**) Effect on NaCl on the germination percentage of *crk* seedlings at 5 days **(B)** and 6 days **(C)** after stratification. Values represent mean of the ratio ± SE (germination percentage on 120 mM NaCl / germination percentage on control medium) for each line (*n* = 15). Asterisks indicate differences between *crk* mutants and Col-0 with statistical significance at *P<0.05, **P<0.01 and ***P<0.001 using Tukey’s HSD *post hoc* test (for **A**) or linear model with single-step p-value adjustment for multiple comparisons (**B** and **C**). Experiments were repeated three times with similar results.
(EPS)

S14 Fig Guard-cell expression of *CRKs*. **(A)** Expression analysis of *CRKs* in guard cell *versus* whole leaf. The publicly available *Arabidopsis* (ecotype: Col-0) microarray data (Agilent *Arabidopsis* Oligo Microarray, Platform ID: GPL9020) was normalized by quantile normalization method. Red and blue indicate increased or decreased gene expression levels in guard cells compared to intact whole leaves, respectively. The intensity of the color is proportional to the absolute value of the fold difference. The solid blue line illustrates the value of the fold change with respect to no fold change (dashed line). The preferentially guard cell expressed genes (*SLAC1* and *KAT1*), mesophyll expressed gene (*CNGC4*) and FLAGELLIN-SENSITIVE 2 (*FLS2*) were included for comparison. **(B)** qPCR analysis of guard cell expression of *CRKs*. Specific *CRKs* are preferentially expressed in guard cells compared to the entire, intact leaf. cDNA was prepared from RNA isolated from guard cell protoplasts isolated after enzymatic digestion to obtain the protoplasts. Control cDNA was prepared from total leaf RNA isolated from untreated leaves. *CRK* transcript abundance was analyzed by qPCR. Expression levels were normalized to five reference genes (*ACT2*, *YLS8*, *PP2AA3*, *TIP41*, *At3g35530*). Data integrated from two independent repeats. *CRK1*, *CRK32*, *CRK33* and *CRK46* were not tested.
(EPS)

S15 Fig Stomatal development and responses are impaired in selected *crks*. **(A)** Stomatal apertures were measured 2 h after ABA treatment. 4 *crk* mutants are impaired in stomatal closure after 2 h of a 10 μM abscisic acid (ABA) treatment. Results are means of % stomatal aperture ratio (width/length) after treatment ± SE (average number of stomata measured = 250). Asterisks indicate statistical significance between control and ABA treatment at *P<0.05, **P<0.01 and ***P<0.001 (linear model, single-step p-value adjustment). Lowercase letters indicate statistical significance between wild type Col-0 and *crk* mutant at *P*<0.05 (a), *P*<0.01 (b) and *P*<0.001 (c) according to one-way ANOVA with *post hoc* Dunnett’s test. **(B)** Comparison between stomata density (number of stomata/mm^2^) and stomatal length (μm) show a significant correlation between the 2 parameters. Most of the *crks* exhibit a smaller stomata density which correlates with longer stomata. In marginal density plot of stomatal length, two clusters can be identified. Grey dashed line shows regression fit with correlation (R), coefficient of determination (R^2^) and significance reported in lower right corner. Results are means (average number of stomata measured = 500). The experiment was repeated three times with similar results.
(EPS)

S16 Fig Stomatal response of *crk* mutants. **(A)** Steady-state stomatal conductance for the *crk* mutants. In case of several alleles an average is shown. Red and blue indicate increased or decreased measures with respect to Col-0, respectively. The intensity of colours is proportional to the Benjamini-Hochberg false discovery rate (FDR) adjusted Z statistic, which takes the estimated means and their variation into account. Roughly, |Z|>2 corresponds to an FDR<5%, and |Z|<2.6 to an FDR<1%. Grey cell = no data. Changes in stomatal conductance (in relative units) in response to 3-min pulse of 500–600 ppb of O_3_ **(B)**, darkness **(C)** and elevating CO_2_ from 400 to 800 ppm **(D)**, calculated as (gst_16_-gst_0_)/gst_0_, where gst_16_ is the value of stomatal conductance 16 min after application of the treatment and gst_0_ is the value of pre-treatment stomatal conductance. Results are means ± SE (*n* = 3–11). Asterisks indicate differences between a *crk* mutant and Col-0 with statistical significance at * *P*<0.05 and ***P<0.001 (ANOVA and Dunnett's test). Experiment was repeated three times with similar results.
(EPS)

S17 Fig Stomatal response of *crk* mutants to O_3_. Time-resolved patterns of stomatal conductance before and after application of four minute pulse of 500–600 ppb of O_3_ at 0 timepoint. Data is presented in relative units normalized to values at 0 timepoint. Results are means ± SE (*n* = 3–11). Asterisks indicate differences between a *crk* mutant and Col-0 with statistical significance at ***P<0.01 at 18 min timepoint (ANOVA and Dunnett's test). The experiment was repeated three times with similar results.
(EPS)

S18 Fig Stomatal response of *crk* mutants to darkness. Time-resolved patterns of stomatal conductance before and after application of artificial darkness at 0 timepoint. Data is presented in relative units normalized to values at 0 timepoint. Results are means ± SE (*n* = 3–11). Asterisks indicate differences between a *crk* mutant and Col-0 with statistical significance at * *P*<0.05 and ***P<0.001 at 16 min timepoint (ANOVA and Dunnett's test). The experiment was repeated three times with similar results.
(EPS)

S19 Fig Stomatal response of *crk* mutants to CO_2_. Time-resolved patterns of stomatal conductance before and after application of 800 ppm of CO_2_ at 0 timepoint. Data is presented in relative units normalized to values at 0 timepoint. Results are means ± SE (*n* = 3–11). Asterisks indicate differences between a *crk* mutant and Col-0 with statistical significance at * *P*<0.05 at 16 min timepoint (ANOVA and Dunnett's test). The experiment was repeated three times with similar results.
(EPS)

S20 Fig Integrated cluster analysis of *crk* mutant stomatal conductance responses. Red and blue indicate increased or decreased measures with respect to Col-0, respectively. The intensity of colours is proportional to the Benjamini-Hochberg false discovery rate (FDR) adjusted Z statistic, which takes the means and their variation into account. Roughly, |Z|>2 corresponds to an FDR<5%, and |Z|<2.6 to an FDR<1%. Hierarchical clustering was carried out with complete linkage algorithm using 1-Pearson correlation as distance.
(EPS)

S21 Fig Basal ROS production in Col-0 wild type and *crk* mutants. Several *crk*s show differences in basal ROS production compared to Col-0 wild type. Lines with significant differences to Col-0 wild type according to Student’s *t*-test with Benjamini-Hochberg false discovery rate correction (P<0.05) are highlighted in blue.
(EPS)

S22 Fig Immunity to hemibiotrophic bacterial pathogens is impaired in *crk* mutants. **(A)** ROS production was enhanced in several *crk* mutants compared to wild type after elicitation by 100 nM flg22 in 4 weeks old leaves. Data show the relative percentage of the mean of the sum of RLU (relative light units) to Col-0 ± SE (*n* = 24). Asterisks indicate differences between *crk* mutants and Col-0 with statistical significance at *P<0.05, **P<0.01 and ***P<0.001 (one-way ANOVA *post hoc* Dunnett). **(B)** Susceptibility to *Pto* DC3000 was increased in several *crk* mutants compared to wild type. *Pto* DC3000 was sprayed onto 2 week-old seedlings at 10^8^ cfu ml^-1^ and disease symptoms were scored 3 days post inoculation using the following 0–3 scale: 0, no symptom; 1, 1 symptomatic cotyledon; 2, 2 symptomatic cotyledons; 3, dead seedling. Results are means ± SE (*n* = 48). Asterisks indicate statistical significance at *P<0.05, **P<0.01 and ***P<0.001 (Mann-Whitney test, Benjamini-Hochberg correction for multiple comparisons). Experiments were repeated three times with similar results.
(EPS)

S23 Fig Stomatal closure is impaired in *crks* in response to flg22 and chitin treatments. **(A)** Stomatal apertures were measured 2 h after flg22 treatment. 12 *crks* mutants are impaired in stomatal closure after 2 h of a 10 mM flagellin (flg22) treatment. Results are means of % stomatal aperture ratio (width/length) after treatment ± SE (average number of stomata measured = 250). Asterisks indicate statistical significance between control treatment at *P<0.05, **P<0.01 and ***P<0.001 (linear model, single-step p-value adjustment). **(B)** Stomatal apertures were measured 2 h after chitin treatment. Several *crk* mutants are impaired in stomatal closure after 2 h of a 1 g.l^-1^ chitin treatment. Results are means of % stomatal aperture ratio (width/length) after treatment ± SE (average number of stomata measured = 250). Asterisks indicate statistical significance between control treatment at *P<0.05, **P<0.01 and ***P<0.001 (linear model, single-step p-value adjustment). Experiments were repeated three times with similar results.
(EPS)

S24 Fig Defense against fungal pathogens is impaired in *crk* mutants. **(A)** Transcript levels of *CRKs* in Col-0 in response to infection with *Go* or *Bgh* over three time points coinciding with fungal penetration and early colonization. The numbers of fungal-responsive *CRK* transcripts increased at later time points. The experiment was conducted three times; Venn diagram shows significantly altered *CRK* transcripts compared to uninfected control plants according to Student’s *t*-test (*P*<0.05). *CRK1*, *12*, *23*, *25*, *33* and *46* were not tested. **(B)** Relative amount of plant foliar coverage with the virulent biotrophic powdery mildew fungal pathogen *Golovinomyces orontii* (*Go*) on Col-0, the *crk* mutant lines and the super susceptible *eds1* mutant. Five plants of each line were scored for percentage *Go* cover by analysis of digital images using the processing software ImageJ (http://imagej.nih.gov/ij/). The experiment was conducted three times and the amount of disease was normalized between experiments by setting the infection cover of Col-0 to one. Error bars indicate ± SD. Asterisks indicate differences between *crk* mutants and Col-0 with statistical significance at *P<0.05, **P<0.01 and ***P<0.001 (linear model, Benjamini-Hochberg false discovery rate adjustment). **(C)** Pictures of *Golovinomyces orontii* (*Go*) infected *crks* and close-up of infected leaves, and on super *Go*-susceptible *eds1*. In some cases, infected leaves displayed increased pigmentation and *crk5* showed accelerated death of the infected leaves. Bar = 1 cm.
(TIF)

S25 Fig Integrated cluster analysis of *crk* mutant phenotypes. An age-matched collection of T-DNA insertion lines in *CRK* genes was analysed for developmental and stress-related phenotypes. Analysis of developmental phenotypes of *crk* mutant lines: endosperm rupture, senescence, epidermal cell segmentation, bolting, flowering, and root length. Analysis of abiotic stress phenotypes of *crk* lines: germination on NaCl medium and analysis of cell death (measured by electrolyte leakage) in response to ozone (O_3_), Xanthine-Xanthine Oxidase (X+XO), ultraviolet radiation (UV-A and-B) or light stress. Analysis of photosynthesis responses upon treatment with 3-(3,4-dichlorophenyl)-1,1-dimethylurea (DCMU) or methyl viologen (MV). Analysis of pathogen susceptibility: analysis of ROS production in response to treatment with bacterial flagellin (flg22) and *crk* susceptibility to the hemibiotrophic bacterial pathogen *Pseudomonas syringae* pv. *tomato* DC3000 (*Pto* DC3000) or the biotrophic fungal pathogens *Golovinomyces orontii (Go)* (virulent on *Arabidopsis*) or *Blumeria graminis* f.sp. *hordei* (*Bgh*) (a barley pathogen, non-pathogenic on *Arabidopsis*). Analysis of stomatal parameters: water loss, density, length, aperture, stomatal aperture in response to ABA or PAMP (flg22 and chitin) treatments and the change in stomatal conductance 16 min after application of an elevated CO_2_, darkness or O_3_ pulse. Experiments were made comparable by bootstrap sampling to *n* = 15 followed by averaging over bootstrap estimates. Red and blue indicate the Benjamini-Hochberg false discovery rate adjusted level of significance of increased or decreased response with respect to Col-0 measured in terms of adjusted Z scores, respectively (S6 Table shows description of plotted values; Fig 10 shows a plot thresholded by the statistical significance of the response).
(EPS)

S1 Table List of the *crk* T-DNA insertion and control lines used for the study. * = source: T-DNA Express *Arabidopsis* Gene Mapping Tool (http://signal.salk.edu/cgi-bin/tdnaexpress); ** = insertion number has been determined by qPCR (Cf. Supplemental Methods). *** = source: Nottingham Arabidopsis Stock Centre (http://arabidopsis.info/Stock Detail Page); **** = sequences designed using T-DNA Primer Design (http://signal.salk.edu/tdnaprimers.2.html). LbaI primer sequences used for: SALK lines: TGGTTCACGTAGTGGGCCATCG except for lines: *crk31*/*36*/*37*/*38*/*39*/*40* ATTTTGCCGATTTCGGAAC WiscDsLox Lines: AACGTCCGCAATGTGTTATTAAGTTGTC SAIL lines: TTCATAACCAATCTCGATACAC
(XLSX)

S2 Table Summary of the stresses and developmental/growth parameters that were used to investigate the *crk* mutant collection. (XLSX)

S3 Table Endosperm rupture. The table shows time course analysis of endosperm rupture of *crks*. This phase of seed germination was monitored at the indicated time periods, every 5 hours. The incubation was in continuous light at 20°C. Mean values of seed germination percentages from three independent experiments are presented ± SE (*n* = 30). Asterisks indicate differences between a *crk* mutant and Col-0 according to one-way-ANOVA (*post hoc* Dunnett, asterisks indicate statistical significance at *P<0.05, **P<0.01 and ***P<0.001).
(XLSX)

S4 Table Fresh weight. Whole rosette from three old-week seedlings were excised and weighted during 4 h. Results are means of fresh weight loss expressed in percentage ± SE (*n* = 5, three independent experiments). Asterisks indicate differences between a *crk* mutant and Col-0 according to one-way-ANOVA (*post hoc* Tukey HSD, asterisks indicate statistical significance at *P<0.05, **P<0.01 and ***P<0.001).
(XLSX)

S5 Table Tissue expression of CRKs according to eFP browser. (XLSX)

S6 Table Summary of the modelling parameters for data analysis. (XLSX)
