# Supplementary figures and images for: Large-Scale Phenomics Identifies Primary and Fine-Tuning Roles for CRKs in Responses Related to Oxidative Stress
Source: PLoS Genet. 2015 Jul 21;11(7):e1005373. doi: 10.1371/journal.pgen.1005373 (PMC4511522; doi:10.1371/journal.pgen.1005373)

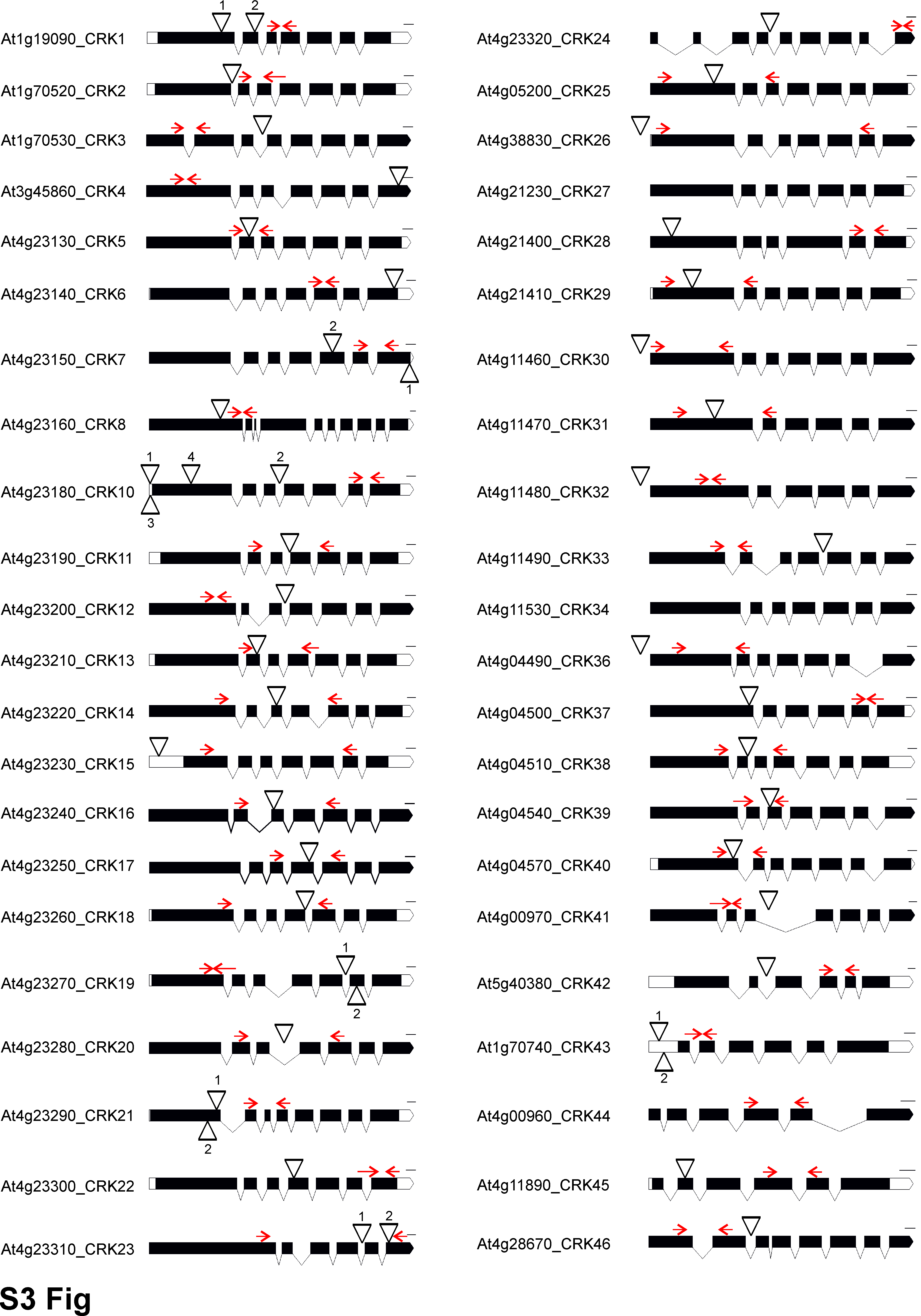

Supplement: S3 Fig — CRK UTR regions, exons and introns have been drawn using the application Exon-Intron Graphic Maker (http://wormweb.org/exonintron). Open triangles indicate the position of the T-DNA insertion. Numbers indicate the different alleles used for the crk lines (crk1-1/2, crk7-1/2, crk10-1/2/3/4, crk19-1/2, crk21-1/2, crk23-1/2, and crk43-1/2). Arrows indicate primer positions used for RT-PCR. The putative pseudogene CRK35 (At4g11500; DUF26 44) and the truncated CRK9 (At4g23170) were excluded from this collection. No homozygous T-DNA insertion lines in Col-0 background were obtained for CRK27, CRK34 and CRK44. Bar = 100 bp. (TIF) [file pgen.1005373.s003.tif]

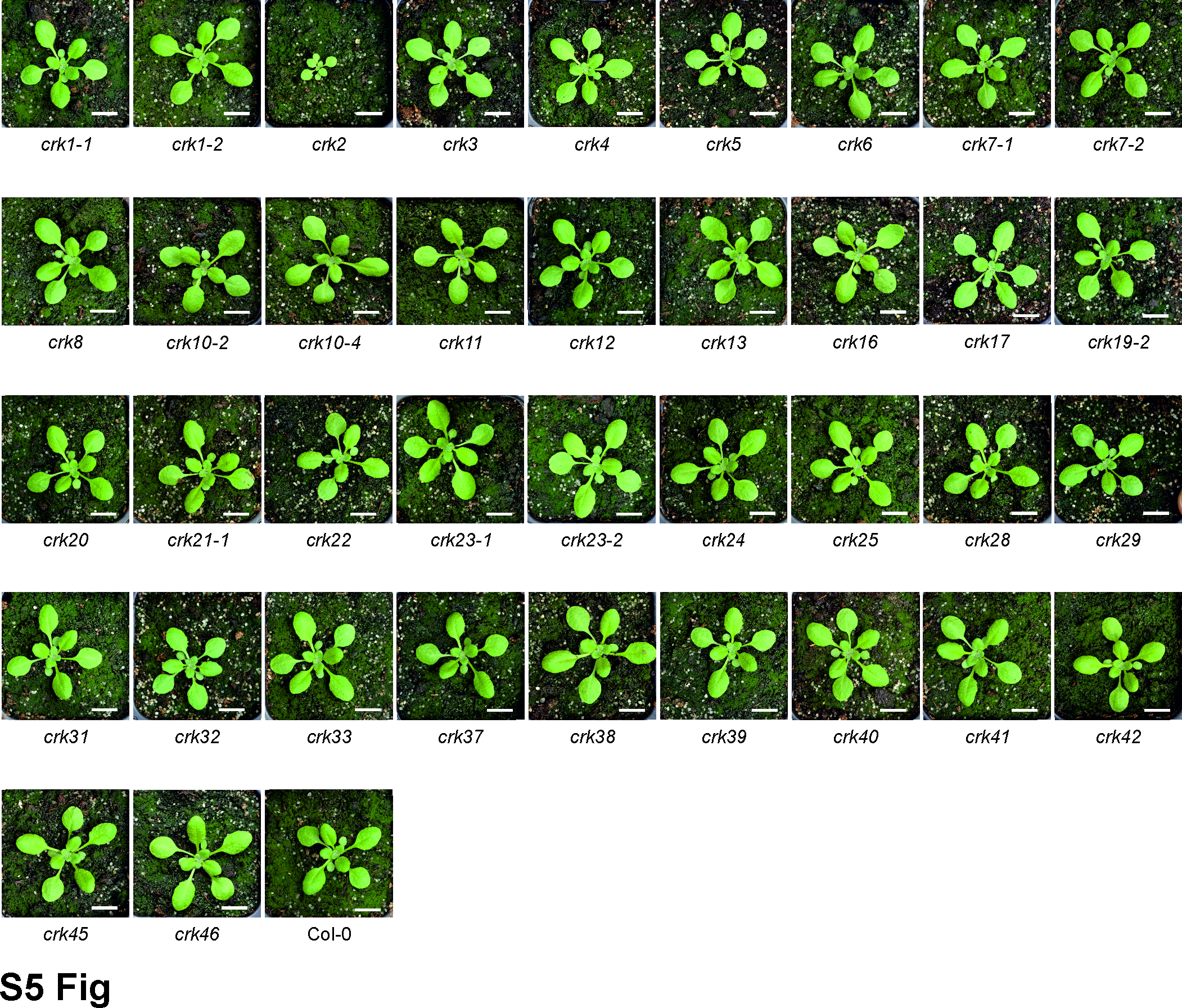

Supplement: S5 Fig — Plants were grown under the following conditions: 250 μmol m-2 s-1 under 12 h-day length (day: 23°C, 70% relative humidity; night: 18°C, 90% relative humidity). Bar = 1 cm. Pictures are representative of three independent experiments. (TIF) [file pgen.1005373.s005.tif]

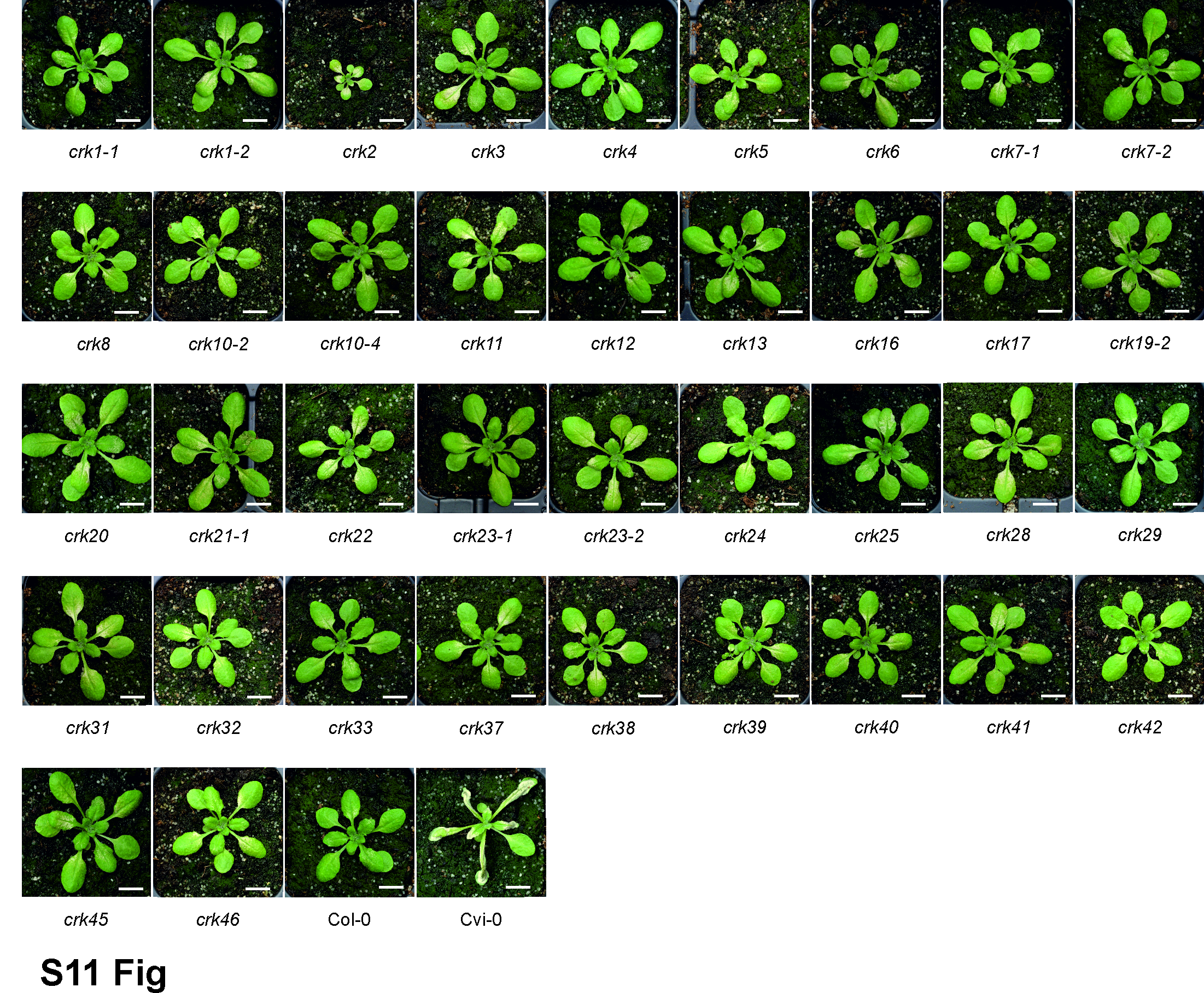

Supplement: S11 Fig — Representative pictures of O3-induced damage in crk seedlings observed at 32 h after initiation of an O3 fumigation (350 ppb, 6h). Lines were grown under the following conditions: 250 μmol m-2 s-1 under 12 h day length (day: 23°C, 70% relative humidity; night: 18°C, 90% relative humidity) during 17 days. On 18th day, plants were exposed to O3 (350 ppb, during 6 h). Pictures were taken 32 h after the initiation of the O3 fumigation; pictures are representative of two independent experiments (n = 6). Bar = 1cm. (TIF) [file pgen.1005373.s011.tif]

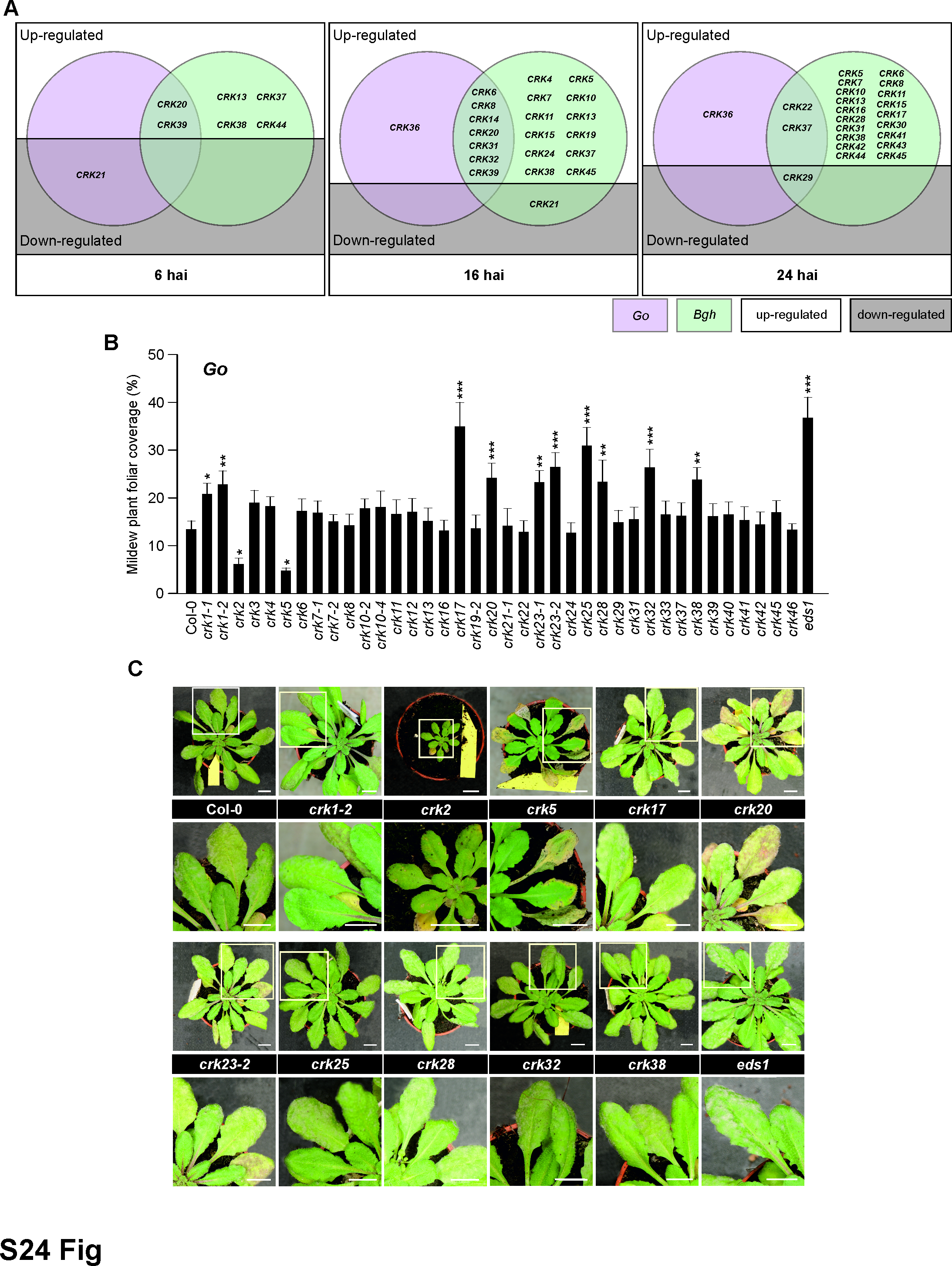

Supplement: S24 Fig — (A) Transcript levels of CRKs in Col-0 in response to infection with Go or Bgh over three time points coinciding with fungal penetration and early colonization. The numbers of fungal-responsive CRK transcripts increased at later time points. The experiment was conducted three times; Venn diagram shows significantly altered CRK transcripts compared to uninfected control plants according to Student’s t-test (P<0.05). CRK1, 12, 23, 25, 33 and 46 were not tested. (B) Relative amount of plant foliar coverage with the virulent biotrophic powdery mildew fungal pathogen Golovinomyces orontii (Go) on Col-0, the crk mutant lines and the super susceptible eds1 mutant. Five plants of each line were scored for percentage Go cover by analysis of digital images using the processing software ImageJ (http://imagej.nih.gov/ij/). The experiment was conducted three times and the amount of disease was normalized between experiments by setting the infection cover of Col-0 to one. Error bars indicate ± SD. Asterisks indicate differences between crk mutants and Col-0 with statistical significance at *P<0.05, **P<0.01 and ***P<0.001 (linear model, Benjamini-Hochberg false discovery rate adjustment). (C) Pictures of Golovinomyces orontii (Go) infected crks and close-up of infected leaves, and on super Go-susceptible eds1. In some cases, infected leaves displayed increased pigmentation and crk5 showed accelerated death of the infected leaves. Bar = 1 cm. (TIF) [file pgen.1005373.s024.tif]
